# Hydrogels—Advanced Polymer Platforms for Drug Delivery

**DOI:** 10.3390/polym18060709

**Published:** 2026-03-14

**Authors:** Rodica Ene (Vatcu), Andreea-Teodora Iacob, Iuliu Fulga, Maria Luisa Di Gioia, Ionut Dragostin, Ana Fulga, Sangram Keshari Samal, Oana-Maria Dragostin

**Affiliations:** 1 Research Centre in the Medical-Pharmaceutical Field, Faculty of Medicine and Pharmacy, “Dunarea de Jos” University of Galati, 35 AL Cuza Street, 800010 Galati, Romania; rodica.vatcu@ugal.ro (R.E.); fulgaiuliu@yahoo.com (I.F.); ana.fulga@ugal.ro (A.F.); oana.dragostin@ugal.ro (O.-M.D.); 2 Department of I Pharmaceutical Science, Faculty of Pharmacy, Grigore T. Popa University of Medicine and Pharmacy Iasi, 700115 Iași, Romania; 3 Dipartimento di Farmacia, Salute e Scienze della Nutrizione, Università della Calabria, Arcavacata di Rende, 87036 Cosenza, Italy; maria_luisa.digioia@unical.it; 4 Laboratory of Biomaterials and Regenerative Medicine for Advanced Therapies, AcSIR Faculty of Medical Research, Indian Council of Medical Research-Regional Medical Research Center, Bhubaneswar 751023, India; sksamalrec@gmail.com

**Keywords:** hydrogels, polymeric platforms, stimuli-responsive, drug delivery, biomedical applications

## Abstract

Optimizing drug administration remains a central challenge in the development of modern therapies, especially in the context of conditions that require spatiotemporal control of active substance release. In this context, hydrogels have been intensively investigated as polymeric platforms for drug delivery, through their three-dimensional hydrophilic structure, tunable properties, and compatibility with biological environments. This analysis presents an integrated approach to hydrogels used in drug administration, addressing the physicochemical fundamentals, the constitutive polymeric materials, and the mechanisms of response to relevant physiological stimuli. Recent experimental studies have been discussed, which highlight the use of hydrogels based on natural, synthetic, and hybrid polymers for controlled and targeted release, in correlation with various administration routes, including oral, injectable, transmucosal, and topical ones. Advanced functionalization strategies that allow adaptive responses to pH, temperature, glucose, enzymes, and reactive oxygen species are also analyzed. Furthermore, emerging directions integrating hydrogels with biosensors, microdevices, and wireless communication systems for real-time monitoring and on-demand release are highlighted. Overall, the analysis emphasizes the role of smart hydrogels as multifunctional platforms for complex therapeutic strategies while also underlining the current challenges associated with clinical translation and long-term performance.

## 1. Introduction

Recent advances in the field of drug delivery systems have highlighted significant benefits of modern drug delivery platforms compared to conventional formulations, including improved bioavailability, control of release kinetics, and reduction in undesired systemic exposure [1]. Therapeutic efficacy largely depends on maintaining effective drug concentrations at the site of action for adequate periods of time, a goal that is difficult to achieve through classical administrations [2]. In this regard, smart delivery systems offer spatiotemporal control, allowing on-demand loading and release of drugs according to local physiological or pathological conditions [3].

Hydrogels stand out as some of the most promising drug carriers due to their hydrophilic three-dimensional structure, which gives them a high capacity for absorbing biological fluids and water, and, at the same time, a structural similarity to living tissues [4]. These materials offer a unique combination of properties useful in drug delivery, such as biocompatibility, biodegradability, and the ability to protect therapeutic molecules from premature degradation in complex biological environments [5]. In addition, hydrogels allow the adjustment of the polymer network by modifying the crosslinking density and the chemical composition, which directly influences the kinetics of drug release [6]. As the field of advanced materials has evolved, simple hydrogels have been gradually replaced by smart hydrogels endowed with stimulus-responsive capabilities [7]. Such hydrogels modify their physicochemical properties, such as the swelling degree, permeability, or interactions with the drug, as a result of the internal stimuli intervention, such as pH, temperature, reactive oxygen species (ROS), or specific enzymes [8,9]. This adaptability enhances the efficiency of drug release and reduces the need for frequent administration, contributing to personalized therapies with improved tolerability. Smart hydrogels are designed to respond to local physiological conditions, providing precise control over therapeutic release, decreasing the undesired accumulation of drugs in normal tissues, and limiting potential adverse effects [10]. This characteristic is especially important in treatments that need prolonged or targeted administration, such as oncological therapies, autoimmune therapies, or local administrations in chronic diseases [11,12,13]. Another important property of hydrogels is their versatility regarding the routes of administration. Due to the possibility of controlled adjustment of their structure and properties, hydrogels can be optimized for oral administration, protecting sensitive drugs from the specific environments of the gastrointestinal tract [14,15]. They can also be formulated as injectable in situ systems that gel at body temperature, providing sustained delivery [16,17]; they can be adapted for transmucosal or topical administration with prolonged local retention [18,19,20], as well as for other complex therapeutic strategies [21]. Furthermore, smart hydrogels can be associated with emerging technologies, such as biosensors, wireless modules, and biomedical feedback systems, which allow continuous monitoring of biomarkers and “real-time” drug release based on physiological signals, paving the way for adaptive and personalized therapies [22,23,24]. Moreover, by integrating advanced functional elements, these systems go beyond the classical paradigm of stimuli-responsive hydrogels and evolve toward interactive platforms capable of correlating local biomarker detection with adaptive regulation of therapeutic release. The combination of hydrogel matrices with biosensors and monitoring microtechnologies enables the generation of dynamic responses to changes in the pathological microenvironment, while the integration of computational strategies and data analysis algorithms is exploited as an emerging direction for optimizing therapeutic decision-making and for the development of personalized, closed-loop feedback-based drug delivery systems [25,26,27]. Although smart hydrogels have made significant advances in recent years, major challenges remain, including the variability of biological environments, precise control of polymer degradation, and maintaining drug stability within the hydrogel network, as well as scalability issues, long-term biocompatibility, and clinical regulations that need to be solved before large-scale implementation [28].

In this context, the aim of this article is to provide a comprehensive and integrative review of hydrogels as smart polymeric platforms for drug delivery, based on evidence from numerous preclinical and clinical studies. The article synthesizes concrete examples of hydrogels used for various pathologies and administration routes, highlighting how polymer networks, adaptive functionalization, and stimulus-responsive properties contribute to controlled release and enhanced therapeutic efficacy. The approach adopted allows for a holistic perspective on the applicability of hydrogels, focusing particularly on functional performance in real biological contexts and the clinical impact of these systems. Thus, the article offers added value compared to existing reviews by integrating recent preclinical and clinical studies, providing readers with a complete picture of the potential of smart hydrogels in drug delivery and personalized therapies.

## 2. Methodology

For this work, a bibliographic search was conducted in the PubMed (https://pubmed.ncbi.nlm.nih.gov, accessed between 1 July 2025–4 March 2026), Scopus (https://www.scopus.com, accessed between 1 July 2025–4 March 2026), and Web of Science (https://www.webofscience.com, accessed between 1 July 2025–4 March 2026) databases, targeting relevant publications regarding polymeric hydrogels used in drug delivery systems (DDSs). Publications from 1998 to 2026 were considered, and the main keywords (“drug delivery”, “hydrogel”, “polysaccharide-based”, “bio-based hydrogel”, “biomedical”) were strategically combined with Boolean operators (“AND”, “OR”) to optimize the search precision. Initially, 460 publications were identified, and after removing duplicates, the selection process was carried out in two stages: first, screening the titles and abstracts, then evaluating the full text of potentially eligible studies. Following the application of inclusion and exclusion criteria, 354 publications were retained for the final analysis. Original articles and reviews providing relevant information for the development, characterization, and biomedical applications of polymeric hydrogels were included. Original studies were considered eligible if they reported experimental investigations in vitro, ex vivo, or in vivo regarding hydrogel formulation, stimuli-responsive properties, integration into advanced polymeric platforms, and evaluation via different administration routes. Exclusion criteria included publications not relevant to the study objectives, articles in languages other than English, abstracts without full text, and works lacking experimental or conceptual relevance.

## 3. Synthesis, Essential and Specific Properties of Hydrogels

This chapter details the fundamental properties of hydrogels, including swelling capacity, molecular diffusion, biocompatibility, and biodegradability, as well as the relationship between polymer network structure and drug delivery function. Additionally, the main synthesis strategies, the three-dimensional architecture of the network, and the compositional variety of hydrogels, elements that directly influence the material’s behavior and its performance in biomedical applications, are discussed. Key characterization parameters, such as pore size, crosslinking degree, and elasticity, which allow the design of hydrogels with controlled performance, adapted to clinical applications, will also be described.

By understanding these scientific foundations, the design of hydrogels for drug delivery can be optimized, providing efficient, safe, and customizable platforms for innovative pharmacological treatments.

### 3.1. Synthesis, Architecture, and Compositional Aspects

Hydrogels are three-dimensional polymer networks capable of absorbing and retaining large amounts of water or biological fluids. Their hydration capacity, associated with structural flexibility and adaptability to variable environments, confers unique properties that have facilitated significant advances in the biomedical field, including drug delivery, tissue engineering, and regenerative medicine [29,30]. Their physicochemical and mechanical properties are determined both by the type of polymers used, and by the ratio of constituents, composition, and synthesis processes [31]. Functional groups on the polymer chains, such as carboxyl, amino, and hydroxyl, regulate hydrophilicity, swelling behavior, and interactions with biological molecules, with the choice and functionalization of polymers in hydrogel synthesis, influencing their performance and biomedical applications [32]. In addition, the chemical composition of hydrogels influences biocompatibility and biodegradability in biological environments, crucial characteristics for the biomedical field [33]. The synthesis strategy and the three-dimensional architecture of the polymer network influence the crosslinking density, the degree of interconnection of polymer chains, and the network mesh size, parameters that determine swelling capacity, molecular diffusion, and the mechanical properties of the material [34,35]. Hydrogels can be obtained through physical or chemical crosslinking, each method generating structural particularities that determine the characteristic properties of the final material [36,37].

**Figure 1:**
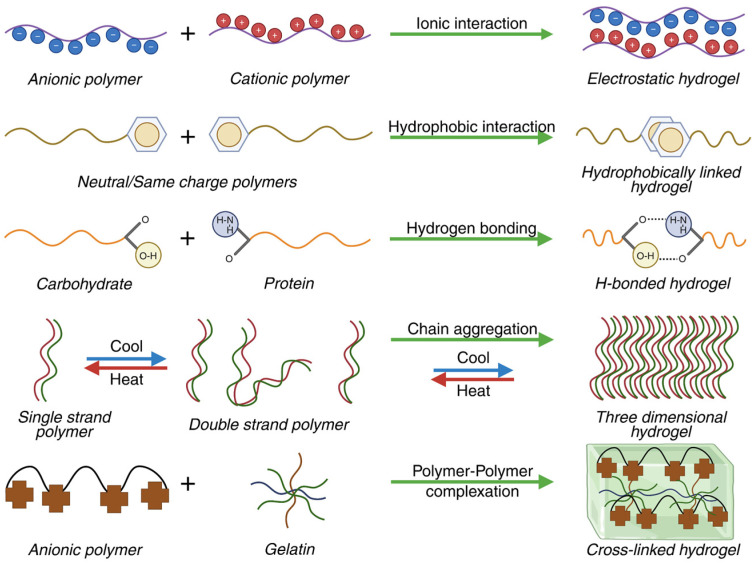
Interaction mechanisms in physical hydrogels. Adapted from ref. [37] and created in BioRender. https://BioRender.com/rnwzlwk (accessed on 21 February 2026) is licensed under CC BY 4.0.

Physically crosslinked hydrogels are three-dimensional networks stabilized by noncovalent interactions between polymer chains, which confer a reversible character and the ability to respond to external stimuli [37,38]. The properties of physically crosslinked hydrogels depend on parameters such as polymer concentration, pH, and temperature, factors that influence the density of association points and the mechanical behavior of the network [39]. This approach is advantageous because it generally eliminates the need for chemical crosslinking agents or initiators, thus reducing potential toxicity and simplifying the synthesis process [40]. Physical crosslinking can be achieved through various mechanisms, such as cryogelation, ionic interactions, hydrogen bonding, stereocomplexation, or thermal aggregation, each method contributing to the stability and behavior of the hydrogels [29,36]. Cryogelation, through the formation of microcrystals, stabilizes the three-dimensional network and influences the porosity and elasticity of the material [41]. Ionic interactions and ionotropic gelation create nodes between polymer chains, regulating crosslinking density, swelling, and molecular diffusion [42,43,44]. In addition, hydrogen bonds ensure mechanical strength, self-healing capacity, and adaptability of hydrogels to variations in pH and temperature [45,46,47,48], while stereocomplexation and thermal aggregation generate additional physical crosslinking points, increasing network stability and swelling capacity [49,50]. The main mechanisms of physical crosslinking are schematically summarized in Figure 1.

**Figure 2:**
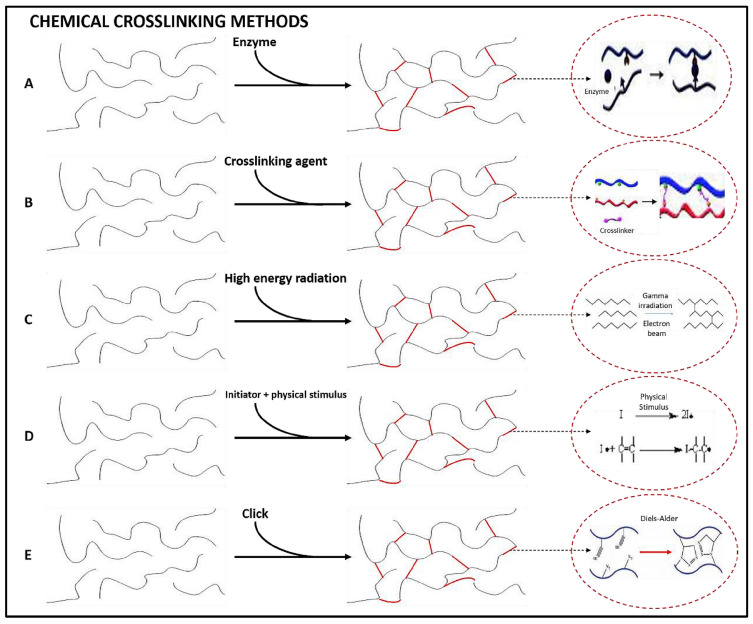
Schematic representation of the mechanisms for the preparation of chemically cross-linked hydrogels: (**A**) enzymatic cross-linking; (**B**) crosslinking using chemical agents; (**C**) high-energy radiation; (**D**) initiator with physical stimulus; (**E**) click chemistry. Reprinted from Sánchez-Cid P. et al., 2022 [4], *Polymers* [CC BY 4.0, https://doi.org/10.3390/polym14153023].

In contrast, chemical crosslinking (Figure 2) involves the formation of covalent bonds between polymer chains, resulting in more stable and mechanically stronger networks, which makes them more suitable for long-term use [51]. Chemical crosslinking can involve enzymatic reactions, chemical reactions promoted by a crosslinking agent, crosslinking with ionizing radiation, or with NIR [4,35].

Enzymatic crosslinking represents a biocompatible method for obtaining hydrogels, based on the use of enzymes as catalytic agents for the formation of intermolecular bonds. This approach contributes to reducing the toxicity associated with conventional chemical crosslinkers, providing milder reaction conditions. In the presence of enzymes, new bonds are generated between polymer chains, leading to the formation of a stable three-dimensional network [4].

**Figure 3:**
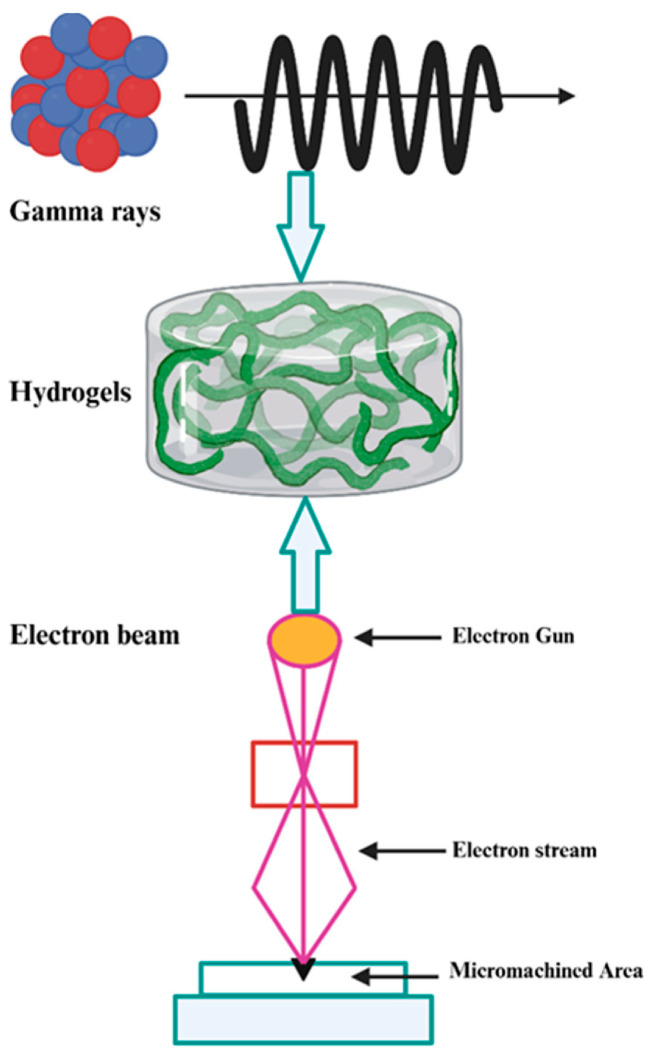
Illustrative scheme of hydrogel synthesis through ionizing radiation. Reprinted from Asim Raza M., 2025 [54], *Gels* [CC BY 4.0, https://doi.org/10.3390/gels11070492].

A modern method for hydrogel synthesis involves chemical crosslinking through near-infrared (NIR) light, which allows the direct formation of the three-dimensional network without the need for traditional crosslinking agents. This strategy provides spatiotemporal control over gelation and produces hydrogels with a stable, porous, and biocompatible structure. The obtained hydrogels can be used as functional platforms for biomedical applications or for the integration of bioactive molecules [52]. For example, Karami et al. [53] used upconversion nanoparticles integrated into a hydrogel matrix to convert NIR energy into radicals that initiate crosslinks between polymers, leading to the formation of the three-dimensional hydrogel network under mild and controlled conditions. The study demonstrated that adhesive hydrogels can be formed and reinforced under biological tissues through NIR, providing a noninvasive method of controlled polymerization, which allows tuning of the hydrogel’s mechanical and adhesive properties and reduces the need for traditional surgical interventions. The synthesis of hydrogels through ionizing radiation (gamma rays, electron beam) represents an efficient method for obtaining covalent networks without the presence of initiators, resulting in relatively pure hydrogels (Figure 3). The energy of the radiation is absorbed by the polymer system or by water in the medium, generating reactive species that induce the formation of molecular radicals, which, through recombination, lead to the formation of the crosslinked three-dimensional network [54,55].

**Figure 4:**
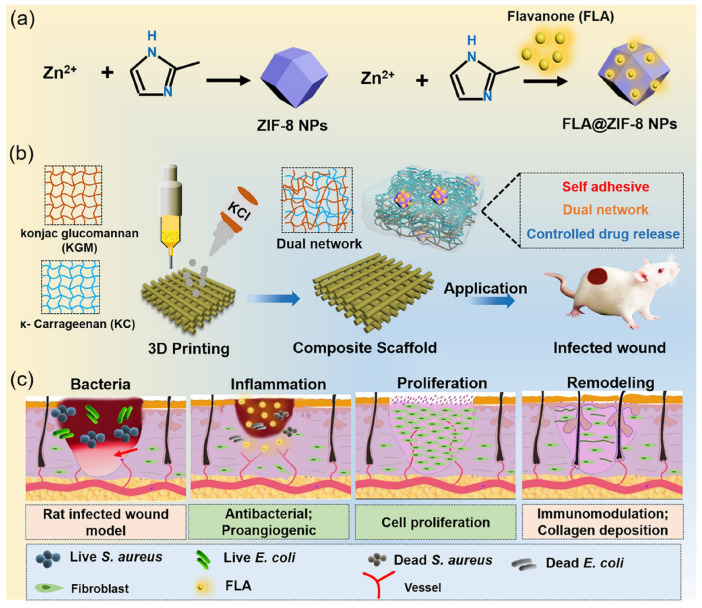
Schematic of the FLA@ZIF-8/KC@KGM hydrogel scaffold and its application in an infected wound model. (**a**) Synthesis of FLA@ZIF-8 nanoparticles. (**b**) Fabrication of the scaffold via 3D bioprinting. (**c**) Effects on wound healing, including controlled drug release and tissue regeneration. The red arrow illustrates the dynamic effect of the FLA@ZIF-8/KC@KGM hydrogel scaffold on tissue repair. Reprinted from Yu J. et al., 2024 [60], *Gels* [CC BY 4.0, https://doi.org/10.3390/gels10120835].

In addition to traditional methods of hydrogel synthesis, an innovative strategy is represented by 3D bioprinting, which allows the creation of complex three-dimensional networks through layer-by-layer deposition of the material, with precise control over network architecture, porosity, and mechanical properties. This technology also facilitates the direct incorporation of functionalized bio-inks, opening new possibilities for biomedical applications, such as scaffolds for tissue regeneration and platforms for controlled delivery of therapeutic agents [55,56,57,58,59]. A recent example is the KC@KGM hydrogel scaffolds with FLA@ZIF-8 nanoparticles fabricated via 3D bioprinting, which ensure controlled release of flavanone and Zn^2+^, effective antibacterial activity, and accelerated healing of infected wounds (Figure 4) [60], demonstrating their potential for advanced biomedical applications.

Depending on the used synthesis method, hydrogels can exhibit varied architectures, from simple homopolymeric or copolymeric structures to semi-interpenetrating (semi-IPN) and interpenetrating (IPN) networks [37].

**Figure 5:**
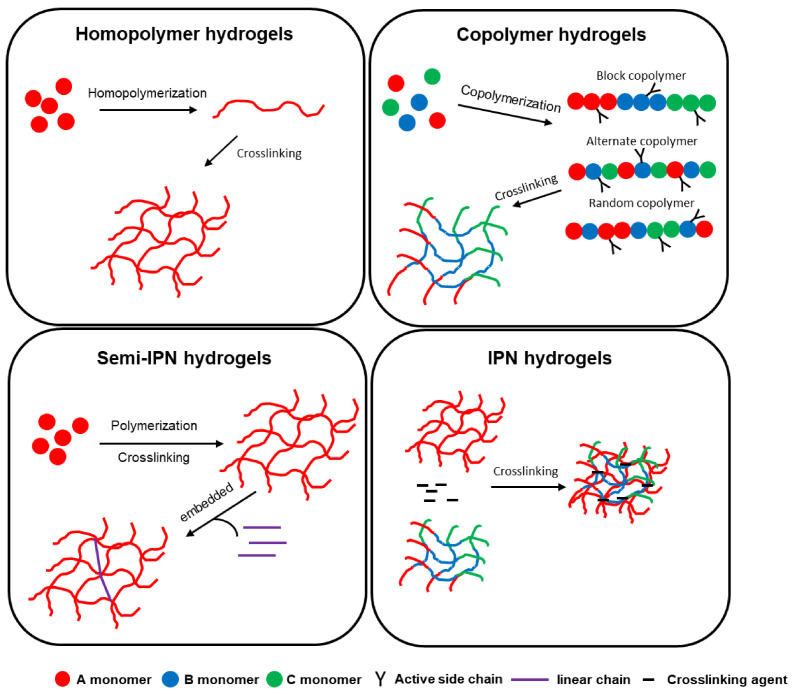
Schematic representation of homopolymer hydrogels, copolymer hydrogels, semi-interpenetrating polymer networks (semi-IPNs), and interpenetrating polymer network (IPN) hydrogels. Reprinted from Ho T.C. et al., 2022 [30], *Molecules* [CC BY 4.0, https://doi.org/10.3390/molecules27092902].

Homopolymeric hydrogels are formed from a single type of monomer, and their properties depend on the nature of the polymer and the method used for crosslinking. These crosslinked networks are frequently used in drug delivery systems and in the production of contact lenses. Among the most used polymers in the synthesis of this type of hydrogel are: polyethylene glycol (PEG), polyvinyl alcohol (PVA), polyvinylpyrrolidone (PVP), and polyacrylic acid (PAA) [61]. In contrast, copolymeric hydrogels combine two or more polymers, either through copolymerization or physical mixing, to adjust physical, chemical, and biological properties. For example, gelatin-alginate combines the cell adhesion of gelatin with the gelation and biocompatibility of alginate, chitosan–hyaluronic acid combines antimicrobial effects with water retention, and collagen–chondroitin sulfate provides structural support and aids cartilage repair [62]. Semi-interpenetrating hydrogels are formed from a primary polymer network partially interlaced with a secondary polymer, without covalent bonds between them. This architecture provides superior mechanical properties, biocompatibility, and functional versatility [63]. For example, in situ-formed collagen–hyaluronate semi-interpenetrating networks have demonstrated efficiency in corneal regeneration by balancing stiffness and biological support [64], while collagen–polyurethane–alginate semi-interpenetrating networks have been investigated for tissue healing applications and controlled drug delivery [65]. Interpenetrating polymer networks (IPNs) are formed from two or more independently crosslinked networks, topologically interconnected at the molecular level. Compared to single-network or semi-interpenetrating hydrogels, these structures exhibit superior mechanical strength and toughness due to efficient stress distribution and limitation of crack propagation [66]. In the literature, IPNs have often been included in the category of composite hydrogels, representing a distinct type of hydrogel with multiple interpenetrating networks, offering significant advantages over conventional composite hydrogels, such as enhanced mechanical strength, increased ductility, improved phase stability, and the possibility of more precise control over swelling behavior and drug release [30,67]. Figure 5 summarizes the classification of composite hydrogels and highlights the position of IPNs in this context.

**Figure 6:**
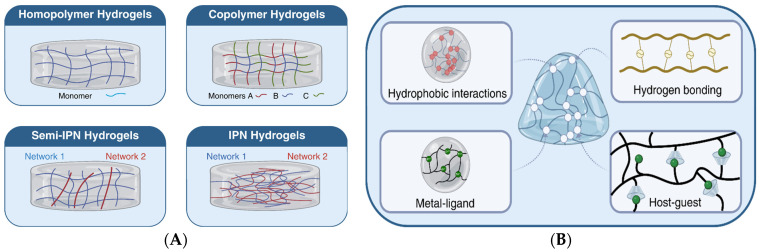
(**A**). Hydrogel Network Architectures: Homopolymer, Copolymer, Semi-IPN, and IPN—created in BioRender. (https://biorender.com/ddfyh5k, accessed on 23 February 2026) is licensed under CC BY 4.0., (**B**). Supramolecular Hydrogels—Representative Interaction Types—created in BioRender. (https://biorender.com/miee9ft, accessed on 23 February 2026) is licensed under CC BY 4.0. The properties and behavior of hydrogels, which will be detailed in the following sections, depend in a determining manner on the polymeric composition of the constituents, which may include natural polymers, synthetic polymers, or combinations thereof. Natural polymers are represented by polysaccharides of plant, animal, or microbial origin and are highly suitable for the preparation of hydrogels intended for the biomedical field due to their excellent properties, such as hydrophilicity, non-toxicity, biocompatibility, and biodegradability [71]. In addition, they possess high swelling capacity and represent important synthesis resources due to their renewability [35,72]. In addition to natural and synthetic polymers, which are discussed extensively in the following section of this article, hydrogels may also include inorganic or nanocomposite materials, such as metallic or oxide nanoparticles, carbon nanotubes, and nanofibers, which contribute to the modification of the mechanical, physicochemical, and functional properties of the network [73,74,75,76].

In addition to composite and interpenetrating networks, research has also focused on supramolecular hydrogels, which are formed through host–guest interactions, electrostatic interactions, metal–ligand binding, and hydrophobic forces [68]. These structures confer dynamic and adaptable properties, such as self-healing and mechanical strength [69]. For example, Fang et al. developed a cyclodextrin-based supramolecular self-healing hydrogel, capable of recovering after mechanical damage, suitable for applications requiring long-term stability and durability [70]. To provide an integrated perspective on the various architectures of hybrid hydrogels, Figure 6A,B summarizes the composite, interpenetrating, and supramolecular structures.

### 3.2. Swelling Behavior

The swelling behavior of hydrogels represents a strategic parameter in determining their performance as controlled drug delivery systems. In aqueous environments, the crosslinked polymer network permits solvent penetration, resulting in volume expansion and relaxation of polymer chains, a process governed by the balance between osmotic forces generated by hydrophilic groups and the elastic constraints imposed by crosslinking bonds [2,77]. Thus, The Flory–Rehner model is the theoretical foundation for understanding the swelling behavior of hydrophilic polymer networks and is discussed in detail in their work by Zang K.et al. This thermodynamic relationship describes the balance between the osmotic forces that favor solvent absorption and the elastic constraints imposed by chemical crosslinks. The chemical potential of the solvent in the gel can be expressed as the sum of the mixing (Flory–Huggins) and elastic contributions, providing quantitative predictions for the degree of swelling at equilibrium as a function of the crosslink density [78].

Thus, the three-dimensional structure of the polymer matrix constitutes a determining factor for the degree and kinetics of swelling [79]. Polymer chains form interstitial domains that facilitate water absorption, while hydrophilic functional groups (−OH, −NH_2_, and −COOH) interact with solvent molecules through hydrogen bonding, promoting hydration and stabilizing the matrix [31]. At the same time, hydrophobic segments and electrostatic interactions between polymer units modulate chain expansion, network permeability, and internal mobility, influencing the diffusion of therapeutic molecules [77,80]. Therefore, swelling behavior depends on the chemical nature of the polymer, crosslinking density, and the ratio of hydrophilic to hydrophobic segments [31]. The crosslinking strategy used in the manufacturing process plays a central role in controlling insolubility, mechanical stability, and structural integrity of the hydrogel during swelling [81]. The polymer–solvent interaction parameter (χ) plays a crucial role in determining thermodynamic compatibility, while the effective crosslink density (νe) controls the elastic response of the network. For pH- or temperature-sensitive hydrogels, changes in χ induced by external stimuli generate dramatic volumetric transitions, with potential for applications in controlled release.

In this regard, the Peppas–Sahlin model provides a description of the release kinetics of active substances from hydrogel matrices, breaking down the diffusional (Fickian) and relaxational (non-Fickian) contributions. This two-component approach is essential for understanding the transport mechanisms in vitrified or semi-crystalline polymers, where the relaxation processes of the macromolecular chains significantly influence the release dynamics. Crosslink density dramatically modulates the balance between these two mechanisms. As the density of crosslink points increases, chain mobility decreases, and cooperative relaxations of macromolecular segments become increasingly limited. This mechanistic transition has profound implications for the design of controlled-release systems tailored to specific biomedical applications, thus requiring optimization of the network architecture for desired release profiles [82,83].

On the other hand, an increased degree of hydration leads to the enlargement of polymer network mesh sizes, facilitating the diffusion and controlled release of encapsulated substances. This property can be modulated by external factors such as pH, temperature, or ionic strength, which induce reversible changes in the internal architecture of the network [3,80]. In the case of oral administration, the pH of the environment influences the ionization degree of functional groups, causing variations in swelling behavior and, consequently, in drug release. Suhail et al. [84] demonstrated that a gelatin–acrylic acid-based hydrogel exhibits pH-dependent swelling, favoring the release of quinine in the intestinal environment (pH 7.4) compared to acidic gastric media. Adjusting polymer and crosslinker concentrations allowed optimization of porosity and swelling kinetics, highlighting the importance of these parameters in designing oral hydrogels with targeted and controlled release [85].

### 3.3. Diffusion in Hydrogels

The release of active compounds from hydrogels depends on the complex interaction between the polymer network architecture and the transport mechanisms of the molecules. In networks with varying degrees of crosslinking, pore size, bond density, and network hydration dictate the mobility of encapsulated molecules [86]. Thus, a well-hydrated network with large pores facilitates rapid diffusion of small molecules, whereas a densely crosslinked structure limits their mobility, favoring prolonged and controlled release. For example, in a recently reported hybrid system, letrozole incorporated into a pHEMA hydrogel combined with PLGA particles was released over a period of 32 days, showing reduced “burst release” and a uniform release rate according to the Fickian diffusion model [87]. From a theoretical perspective, the models used to describe the diffusion of dissolved molecules in hydrogels highlight the differences between homogeneous and heterogeneous structures, quantifying the impact of network architecture on the mobility of the transported species. In the case of homogeneous hydrogels, the hydrodynamic scaling model proposed by Cukier is considered the most consistent with experimental data and with the physical foundation of the parameters involved. However, the model includes a polymer–solvent interaction parameter that is not rigorously defined. Models based on free volume theory are frequently applied under conditions that do not comply with the initial derivation assumptions, which limits their physical consistency. For heterogeneous hydrogels, obstruction-type models show the best agreement with the reported experimental results, while the model proposed by Amsden is considered suitable for describing the influence of polymer chain flexibility on diffusion. The set of these experimental observations highlights the interdependence between the swelling process and the diffusion phenomenon, the hydrogel structure modulating the transport of loaded molecules within the polymeric network [88]. However, drug release is not always limited to a purely diffusion-based mechanism. When the hydrogel network undergoes volumetric changes, such as swelling or polymer chain relaxation, the transport of active substances becomes a mixed process, combining diffusion with the structural mobility of the network. Moreover, the chemical composition of the polymers and the crosslinking density remain key factors in regulating diffusion. PEG-based hydrogels offer extensive customization possibilities by adjusting PEGDA concentration and network density, making it possible to control both the flux and diffusion coefficient of molecules, including drugs such as vancomycin, an antimicrobial compound known for poor absorption and rapid elimination. In this context, greater gel rigidity has been associated with a slower release rate, highlighting the critical role of hydrogel structure in shaping diffusion [89]. Additionally, external factors such as temperature, pH, and light can modulate diffusion, providing opportunities for adaptive control of drug release according to the characteristics of the biological environment [90]. Overall, hydrogels are distinguished by their ability to precisely regulate the diffusion of encapsulated molecules, thanks to the structural flexibility offered by parameters such as crosslinking density, polymer type, swelling capacity, and stimulus responsiveness. This flexibility makes hydrogels predictable and efficient drug delivery systems, capable of optimizing therapeutic profiles and accurately meeting the requirements of controlled-release formulations [91].

### 3.4. Biocompatibility

Biocompatibility represents one of the fundamental properties that determine the performance of hydrogels in therapeutic applications. It reflects the matrix’s capacity to interact with tissues and organs without causing adverse reactions, such as inflammation, unwanted immune responses, or toxicity. For hydrogels used in controlled drug delivery or implantable systems, biocompatibility is essential, as therapeutic success depends not only on the efficiency of active substance release but also on the tolerability within the biological environment. Factors influencing biocompatibility include polymer composition, hydrogel morphology, degree of crosslinking, and the presence of chemical residues (monomers, oligomers, crosslinking agents). Natural polymers, such as chitosan, alginate, gelatin, fibroin, or hyaluronic acid, generally exhibit high biocompatibility and are often preferred in formulations intended for biomedical applications. Although in vitro studies have demonstrated excellent cell viability properties and reduced immune response, in vivo validation remains essential to confirm both the safety and efficacy of hydrogels in clinical applications [92]. A gelatin-based hydrogel demonstrated high biocompatibility in preclinical in vivo studies, without eliciting a significant inflammatory response, highlighting its safety potential for injectable drug delivery [93]. Another injectable hydrogel synthesized from hyaluronic acid and gelatin showed in vivo biocompatibility, supporting cell proliferation and promoting angiogenesis. Preclinical tests indicated that the hydrogel was well tolerated, with no significant inflammatory infiltrates, while also inducing local vascularization, underscoring its potential in regenerative applications [94]. Although natural biopolymers are generally well tolerated by the body, some may trigger immune responses. For example, materials derived from animal sources, such as collagen or gelatin, can cause allergic or inflammatory reactions in certain cases due to residual proteins or endotoxins. Additionally, the use of chemical crosslinking agents or bioactive molecules can enhance cytotoxicity or immunogenic reactions, emphasizing the need for rigorous evaluation of biological compatibility [95,96].

### 3.5. Biodegradability

Biodegradability represents another crucial characteristic of hydrogels used in biomedical applications, being essential for how these materials interact with cells and tissues. Their controlled degradation allows precise modulation of the encapsulated molecules’ release, ensuring continuous and efficient administration in accordance with clinical requirements. This fundamental property has enabled the development of smart materials designed to react to specific stimuli such as pH, temperature, or enzymes. Through this strategy, hydrogels not only maintain matrix integrity until the desired time, but also allow controlled release of drugs or other bioactive factors at the target site. The biodegradation of macromolecules and natural polymers typically occurs through hydrolysis and oxidative processes, which can take place directly in the aqueous environment or be catalyzed by specific enzymes. Biodegradation encompasses all in vivo reactions, whether driven by chemical or metabolic factors. These mechanisms allow the controlled cleavage of chemical bonds within the hydrogel matrix, generating non-toxic degradation products which maintain the material’s biocompatibility. Biodegradable hydrogels have been developed with both kinds of polymers (synthetic and natural), each category offering distinct advantages that influence the material’s properties and applicability. Thus, while synthetic polymers provide high control over physicochemical properties, flexibility in synthesis, and easy commercial availability, natural polymers are preferred for their sustainability, superior biocompatibility, reduced ecological impact, and generation of harmless degradation products [97].

## 4. Polymeric Materials Used in Hydrogel Synthesis

The selection of materials used for hydrogel synthesis is a determining factor in the design of advanced drug delivery systems. The chemical structure, composition, and architecture of the polymer network determine key properties such as swelling degree, permeability, mechanical stability, and biocompatibility, directly influencing release kinetics and drug–matrix interactions. The choice of polymer type, whether natural or synthetic, must be based on the correlation between its intrinsic properties and the intended therapeutic purpose. The release mechanism, whether based on diffusion, degradation, or stimulus responsiveness, as well as the biological compatibility of the material, are critical factors for achieving precise, safe, and efficient administration of active substances.

**Figure 7:**
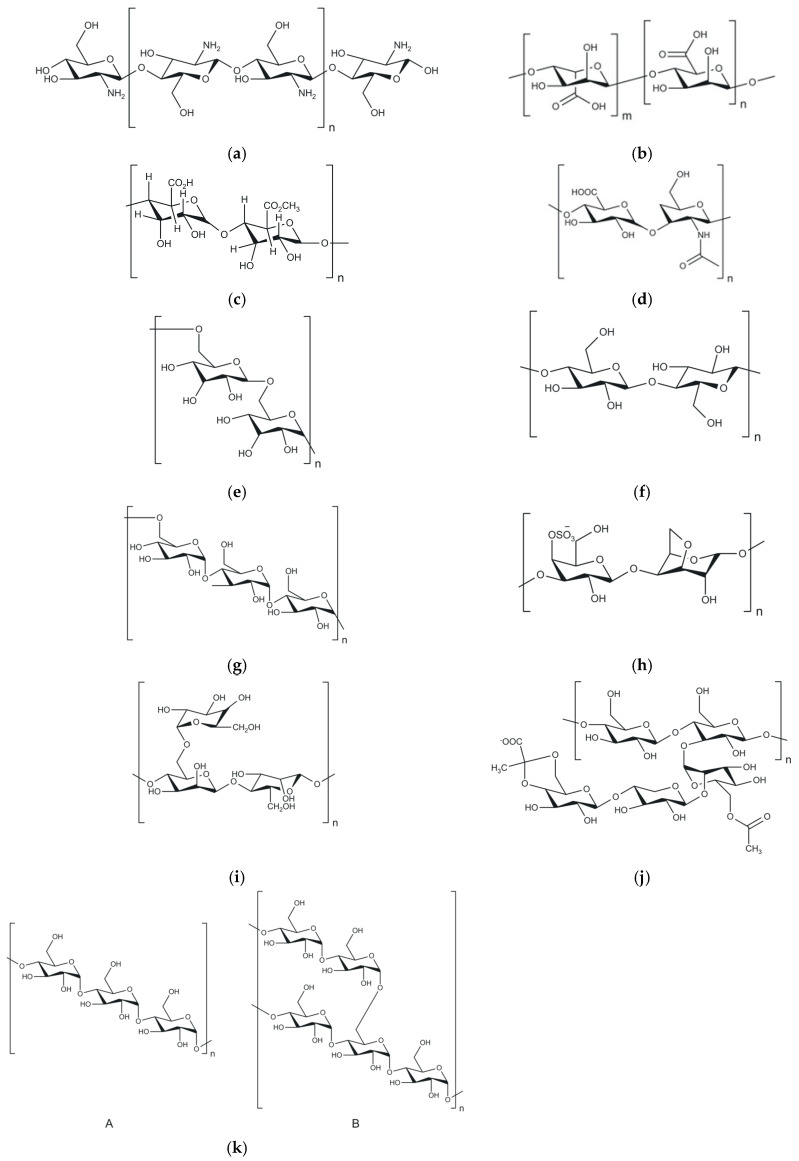
Molecular structure of (**a**) chitosan, (**b**) alginate, (**c**) pectin, (**d**) hyaluronic acid, (**e**) dextran, (**f**) cellulose, (**g**) pullulan, (**h**) κ-carrageenan, (**i**) guar gum, (**j**) xanthan gum, (**k**) starch (A—amylose; B—Amylopectin).

Natural polymers play a key role in the synthesis of hydrogels used for drug delivery, combining biocompatibility, biodegradability, and hydration capacity. Obtained from plant, animal, or microbial sources, these polymers form three-dimensional networks with high water content, which facilitate the adaptive release of bioactive molecules [98]. The presence of hydroxyl, carboxyl, or amino groups allows chemical modifications that adjust network rigidity, crosslinking degree, and responsiveness to specific stimuli, such as pH, temperature, ROS, or the presence of certain biomarkers or enzymes [31]. Structural flexibility and the potential for functionalization make natural polymers suitable candidates for the synthesis of controlled or targeted release networks, applicable via different routes of administration [99]. Within this class, polysaccharides and structural proteins are the most frequently studied materials due to their functional versatility and their ability to form hydrogel networks through distinct mechanisms. Biopolymers such as chitosan, alginate, pectin, hyaluronic acid, dextran, cellulose, or pullulan (Figure 7) can form stable three-dimensional structures through ionic gelation, electrostatic interactions, hydrogen bonding, or covalent crosslinking. For example, alginate is stabilized in the form of hydrogels by interaction with divalent ions, while chitosan exhibits reversible pH-dependent gelation behavior—principles that underpin their use in controlled drug delivery systems [33].

**Figure 8:**
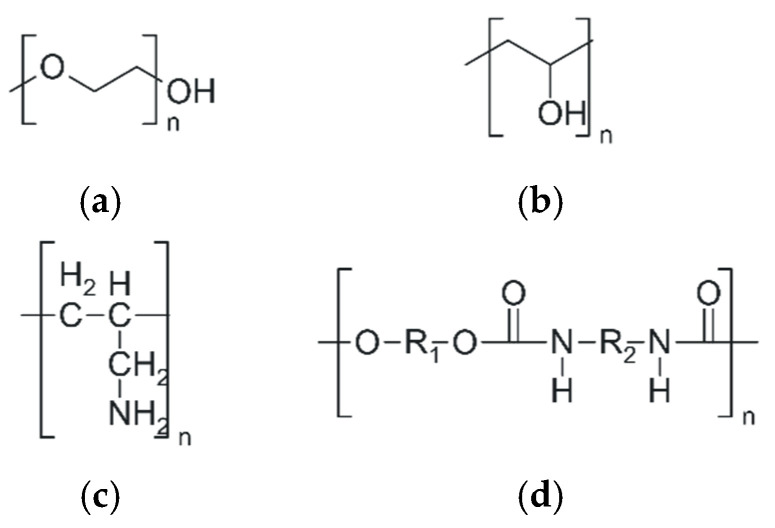
Molecular structure of (**a**) polyethylene glycol, (**b**) polyvinyl alcohol, (**c**) polyacrylamide, (**d**) polyurethane.

Alongside natural polymers, which provide undeniable advantages due to their intrinsic biocompatibility, synthetic polymers represent an essential category of materials used in the design of modern hydrogels for controlled drug delivery (Figure 8). They allow precise molecular engineering of the polymer network, providing control over porosity, degradability, and responsiveness to specific stimuli in the target environment [92]. By manipulating the monomer sequence and selecting the crosslinking method, synthetic polymers can be tailored to produce hydrogels with superior mechanical stability, controlled degradability, and reproducible properties [100]. Due to their structural versatility and potential for chemical functionalization, synthetic polymers generate hydrogels with reproducible properties that can be useful for a wide range of biomedical applications, from drug delivery via various administration routes and targeted therapies to tissue engineering and implantable devices [47,99].

To highlight the scope and diversity of the therapeutic potential of natural and synthetic polymers in formulations for smart drug delivery, Table 1 presents a selection of polysaccharides, structural proteins, and synthetic polymers, along with their functional properties, types of stimulus responsiveness, specific applications, and associated limitations.

Although natural polymers offer significant advantages in hydrogel synthesis, such as biocompatibility and biodegradability, they also present limitations, including low mechanical stability, batch-to-batch variability, and premature degradation in biological environments, while processing or sterilization can affect network integrity [96]. Synthetic polymers guarantee excellent architectural control and reproducibility, but they may be disadvantaged in terms of intrinsic biofunctionality and can generate undesirable systemic effects [124]. The combination of natural and synthetic polymers produces hybrid hydrogels, which merge the advantages of both components, overcoming the limitations of hydrogels synthesized from a single polymer [99]. The literature defines hybrid hydrogels as hydrogel systems obtained by combining components from distinct classes, such as natural and synthetic polymers or polymers combined with inorganic or metallic materials, an association that can generate materials with emergent properties inaccessible to the individual components (Figure 3) [125]. Frequently investigated examples include composite hydrogels, where the integration of nanomaterials provides extended functionalities and control over the release of encapsulated therapeutic compounds; interpenetrating network (IPN) hydrogels, obtained through the synergistic combination of two or more polymer networks; and supramolecular hydrogels, formed through non-covalent interactions between components [66,126,127,128].

Such polymer networks allow the tuning of critical parameters, including mechanical stability, loading capacity, controlled degradation, and responsiveness to physiological or pathological stimuli, as well as the integration of advanced functions such as self-healing, selective transport, or “on-demand” release of bioactive compounds. For example, a composite hydrogel based on carboxymethyl–chitosan (CMCS), graphene oxide, and gelatin demonstrated optimized porosity, improved thermal stability, and pH-dependent controlled release of ibuprofen, with graphene oxide functionalization leading to increased swelling ratio and more efficient control over release kinetics [6].

Interpenetrating network (IPN) hydrogels combine different independent polymer networks within a common matrix, providing enhanced mechanical stability, controlled swelling, and multi-stimuli responsiveness [67]. For example, an IPN hydrogel based on methylcellulose and supramolecular β-cyclodextrin–adamantane networks demonstrated injectability, self-healing, and stimulus-responsive release of diclofenac, showing promise for intra-articular therapies [129]. Similarly, hydrogels composed of microcrystalline cellulose and itaconic acid exhibited pH and glucose sensitivity, protecting insulin in the gastric environment and enabling controlled oral release, maintaining normoglycemia in a diabetic rat model [130]. Moreover, IPN hydrogel microneedles made from silk fibroin and methacrylated hyaluronic acid demonstrated injectability, self-healing, and precise insulin release, effectively regulating blood glucose in diabetic mice [131].

Supramolecular hybrid hydrogels are based on the integration of reversible hydrogen bonding, hydrophobic interactions, metal–ligand coordination, and host–guest chemistry, which enable the formation of dynamic and adaptive networks for controlled drug delivery. These interactions support self-assembly, injectability, and stimulus-dependent release, endowing the hydrogels with emergent properties valuable for advanced drug delivery applications [68]. For example, an injectable supramolecular hydrogel designed for doxorubicin delivery, based on “host–guest interactions” between DOX-2N and β-cyclodextrin, integrated into a Pluronic F-127 and α-cyclodextrin matrix, demonstrated self-healing and pH-dependent controlled release, as shown in ovarian cancer cells [132]. In another approach, an implantable supramolecular hydrogel was developed via Zn(II) coordination with NSAID(MEC) and 5-aminopyridine, forming an injectable, stable polymer network that self-assembles at the implantation site. The system exploits metal–ligand interactions for stability and local retention, providing a versatile platform for controlled drug delivery in oncological applications [133].

Despite their multiple advantages, hybrid hydrogels present common limitations, including variable or low mechanical stability in the absence of further optimization, incomplete or component-dependent biodegradability, differences in solubility of the combined polymers, or the need for specialized crosslinking or processing methods. In addition, the combined materials may exhibit batch-to-batch variations, and the final properties often depend on the proportions and compatibility of the components, requiring rigorous optimization for biomedical applications (Table 2) [99].

## 5. Stimulus-Responsive Smart Hydrogels

**Figure 9:**
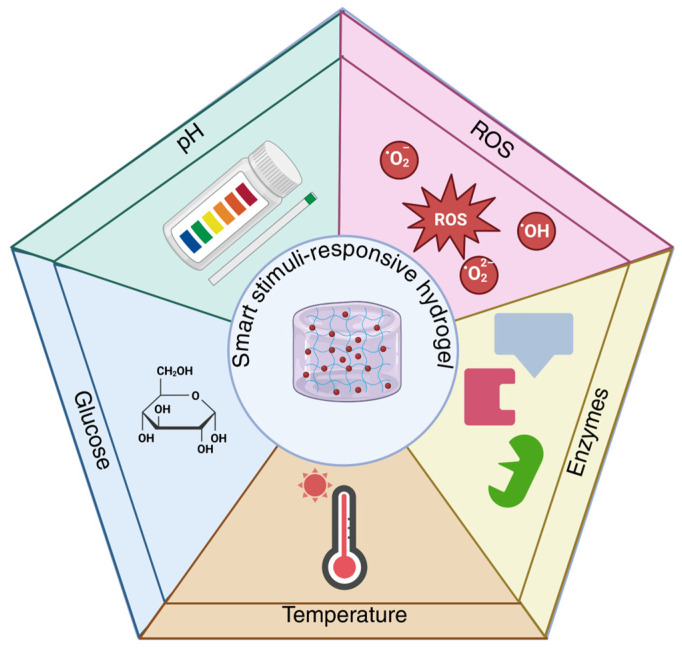
Schematic representation of stimulus-responsive hydrogels, created in BioRender. (https://BioRender.com/o47xoky, 21 December 2025) is licensed under CC BY 4.0.

In the context of precision medicine and the development of personalized therapies, designing drug delivery systems capable of adapting to individual physiological and pathological conditions represents a priority direction in biomedical research. Within this framework, stimulus-responsive hydrogels have attracted particular interest due to their ability to modulate therapeutic release in response to microenvironmental signals, improving treatment efficacy and safety. Unlike conventional hydrogels, which rely predominantly on passive diffusion or degradation mechanisms and have limited capacity for dynamic adaptation to changes in the biological environment, stimulus-responsive hydrogels are designed as intelligent polymeric platforms capable of responding to specific stimuli, thereby overcoming limitations related to tissue specificity [155]. The terms “smart hydrogels” and “stimuli-responsive hydrogels” are used interchangeably in the literature for materials that modify their physicochemical properties in response to external stimuli, such as pH, temperature, light, or electric or magnetic fields [156]. The integration of functional groups sensitive to specific stimuli within the polymer network allows these systems to adjust swelling behavior, porosity, and release kinetics of encapsulated compounds according to the characteristics of the pathological microenvironment [3]. According to the literature, stimulus-responsive hydrogels can be designed to respond to both endogenous stimuli—signals generated internally within the biological microenvironment (Figure 9)—and exogenous stimuli, such as light or electromagnetic fields, applied externally to regulate material properties and therapeutic release [157]. This article focuses on smart hydrogels sensitive to endogenous stimuli, including pH, temperature variations, reactive oxygen species (ROS), specific enzymatic activity, and elevated glucose levels stimuli closely associated with physiological and pathological microenvironments. Harnessing these intrinsic biological signals provides precise drug release control, without the need for externally applied factors.

### 5.1. pH-Sensitive Hydrogels

**Figure 10:**
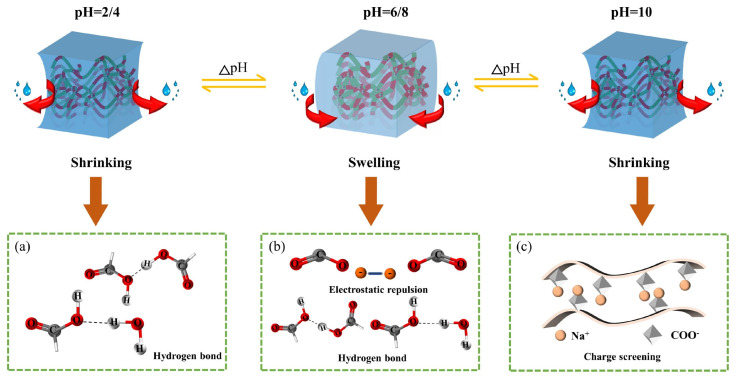
pH-responsive behavior of the hemicellulose-based Fe_3_O_4_@XH-Gel hydrogel. (a) Shrinking at acidic pH (2–4) due to hydrogen bonding between polymer chains. (b) Swelling at near-neutral/basic pH (6–8) due to electrostatic repulsion between ionized polymer chains. (c) Shrinking at basic pH (10) caused by charge screening, which reduces electrostatic repulsion. Reprinted from Jilan Long et al., 2024 [160], *Carbohydrate Polymers* [CC BY 4.0, https://doi.org/10.1016/j.carbpol.2024.122461].

pH-sensitive hydrogels offer the advantage of controlled and targeted drug release in response to variations in the biological environment’s pH, reducing systemic toxicity and enhancing therapeutic potential [158]. The functionality of these systems is based on the ionizable behavior of chemical groups within the polymer network, such that protonation or deprotonation induces reversible changes in volume, porosity, and drug diffusivity [85]. The design of pH-responsive hydrogels involves incorporating chemical components into the polymer network that can respond to pH variations through ionizable functional groups or sensitive chemical bonds, whose ionization or cleavage reversibly or irreversibly alters the hydrogel structure and, consequently, its swelling behavior and drug release [159]. Specific factors, such as local temperature and ionic strength, can also influence the behavior of the ionizable groups [80]. The incorporation of nanoparticles gives the system additional functionalities, such as magnetic reactivity, more precise control of drug release, and increased sensitivity to external factors (Figure 10) [160].

The mechanism of action involves the generation of electrostatic repulsive forces between polymer chains, which leads to reversible swelling and contraction processes dependent on pH, through the absorption or expulsion of water from the porous structure of the hydrogel. Thus, anionic polymers swell at pH > pKa through deprotonation of acidic groups, while cationic polymers swell at pH < pKa through protonation of amino groups, facilitating water penetration and drug release [161].

For example, chitosan, a cationic polymer, swells in acidic environments due to the protonation of amino groups, making it suitable for gastric administration and delivery to areas with pathological acidic pH, such as the tumor microenvironment or diabetic wounds. In contrast, alginate, an anionic polymer, swells at basic pH through ionization of acidic groups, being used for targeted oral administration and drug delivery to the colon [86]. This property of pH-responsive hydrogels can be exploited in oral administration, due to the natural pH variations in the gastrointestinal tract, from 1–3 in the stomach to 7.4–8 in the intestine [162].

pH-responsive hydrogels can also be exploited for targeting the tumor microenvironment, characterized by a slightly acidic pH (approximately 6.5–6.8), compared to healthy tissues (pH approximately 7.4). This difference results from the accelerated glucose metabolism in cancer cells (the Warburg effect), lactate accumulation, and local hypoxia, creating an opportunity for selective drug release [163]. In this context, Liu et al. [164] developed an injectable hydrogel based on glycol–chitosan conjugated with Pluronic F127, capable of forming stable micellar structures and loading doxorubicin through complexation with α-cyclodextrin. The system demonstrated preferential release in the acidic tumor environment, with increased accumulation at the tumor site and reduced distribution to healthy tissues. Compared to free doxorubicin administered intravenously, the hydrogel led to the cessation of tumor proliferation and even partial tumor disappearance.

In addition to gastrointestinal and oncological applications, pH-sensitive hydrogels are also relevant for the management of chronic wounds, where local pH changes constitute an important pathophysiological signal. While healthy skin and acute wounds exhibit a slightly acidic pH (4–6), chronic and infected wounds progressively become more alkaline (pH 7.3–10), as a result of persistent inflammation, hypoxia, and bacterial proliferation [165]. Exploiting this pH variation, Wang et al. designed a biomimetic 3D composite scaffold that integrates an intermediate layer made of polycaprolactone, a pH-sensitive polymer capable of modifying the network structure according to the local environment. The pH-responsive polymer selectively dissolves at alkaline pH values (pH > 7), facilitating network opening and rapid release of encapsulated rifampicin, demonstrating how the ionization of functional groups and conformational changes in the hydrogel network can be exploited to achieve pH-dependent release, tailored to the local pathological state [166].

Although pH-sensitive hydrogels allow targeted and controlled drug release, they present several important limitations. The complex and dynamic variations in pH in the biological microenvironment can lead to unpredictable swelling and release behavior, affecting dosing accuracy. This aspect reflects the fact that the hydrogel response depends both on the polymer properties and crosslinking density, as well as on local pH fluctuations, resulting in non-uniform swelling/deswelling and local drug release [167]. In addition, the stability of pH-responsive hydrogels may be affected by the presence of multivalent ions or in high ionic strength environments, which may lead to premature gelation or accelerated degradation [168]. Despite the advantage of targeted and controlled drug release, their performance remains dependent on factors such as inter-patient pH variability, unforeseen local changes in the tissue microenvironment, and the chemical stability of ionizable groups [85,157]. In addition, the hydrogel response can be affected by interactions with salts, and polymerization to obtain acid-sensitive bonds may raise toxicity issues [1].

### 5.2. Temperature-Sensitive Hydrogels

**Figure 11:**
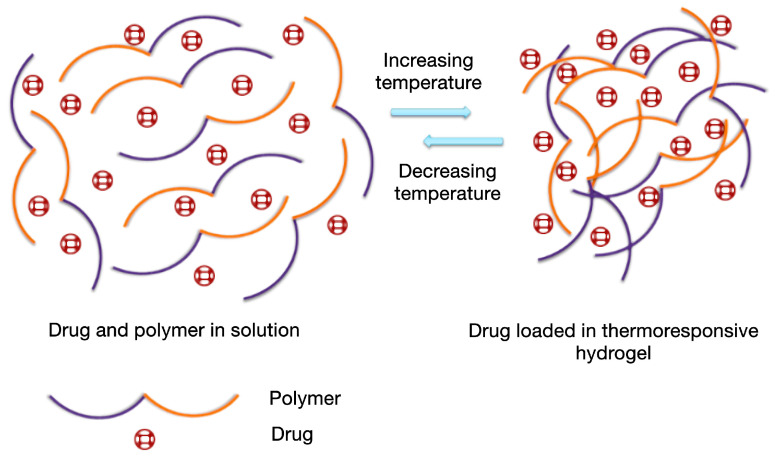
Transition from polymer–drug solution to drug-loaded thermoresponsive hydrogel. Reprinted from Sudipta Chatterjee et al., 2018 [170], *Polymers* [CC BY 4.0, https://doi.org/10.3390/polym10050480].

Thermosensitive smart hydrogels (Figure 11) are polymeric materials capable of responding to temperature variations through reversible sol–gel phase changes, due to amphiphilic polymer chains containing both hydrophilic and hydrophobic segments. This property allows rapid in situ gelation at temperatures close to body temperature, without the need for organic solvents or crosslinking agents [169,170].

The polymers used include both natural polymers, such as chitosan, cellulose derivatives, xyloglucan, or agar, and synthetic polymers, such as poloxamers, poly(N-isopropylacrylamide), and polyethylene glycol [99]. Polymers with a “lower critical solution temperature” (LCST) are particularly promising, gelating near physiological temperature, while polymers with an “upper critical solution temperature” (UCST) do not respond at temperatures close to that of the human body, which limits their biomedical use. Gelation involves the contraction of polymer segments at the LCST, transforming them into hydrophobic and insoluble structures stabilized by hydrophobic interactions and hydrogen bonds. At the same time, the hydrophilic chains absorb water, reinforcing the three-dimensional structure and generating a stable gel with mechanical properties suitable for drug delivery [171].

Injectable thermoresponsive hydrogels have also been evaluated in the oncological context, where systemic administration leads to nonspecific distribution and adverse effects. Formulations such as OncoGel^®^ (PLGA–PEG–PLGA with paclitaxel) and Jelymyto^®^ (UGN-101 with mitomycin) allow direct deposition of the drug at the tumor site, with in situ gelation and prolonged release, increasing local concentration and reducing systemic exposure, which confirms the efficacy and safety of this type of hydrogel in clinical studies [172]. Ophthalmic applications constitute a second relevant domain for thermosensitive hydrogels. Formulations based on alginate–poloxamer 47 stabilized with carboxymethylchitosan have been designed for ocular administration of clobetasol propionate, demonstrating uniform distribution, suitable viscoelastic properties, and sustained drug release. In vivo tests highlighted inflammation improvement and symptom reduction, while in vitro cytotoxicity tests confirmed the excellent biocompatibility of the formulation [173].

Injectable temperature-sensitive hydrogels are also effective in the treatment of skin wounds. An example is the poly(N-isopropylacrylamide) hydrogel conjugated with heparin, loaded with ibuprofen, which gels upon contact with the wound and ensures controlled release of the anti-inflammatory, reducing macrophage activation and promoting tissue repair. The hydrogel’s properties maintain a moist environment, absorb exudates, and accelerate healing in murine models compared to control groups [174].

Overall, these studies proved the applicability of thermosensitive hydrogels in local and targeted drug delivery, confirming the potential of polymers recognized as temperature-sensitive in various therapeutic fields. Despite their advantages, thermo-responsive hydrogels present significant limitations. Precise control of the critical transition temperature (sol–gel) can be affected by local tissue perfusion or in vivo thermal variations, which may lead to premature or delayed gelation and uneven gel formation [175]. In addition, efficient loading of thermolabile drugs and prolonged release are challenging, and the addition of nanoparticles or stabilizing modifiers may raise biocompatibility and safety issues [8]. Other limitations include burst release, limited reproducibility of the sol–gel phase under variable biological conditions, and difficulties in achieving in vivo controlled degradation [172]. These aspects must be considered for the optimization of thermo-responsive formulations prior to clinical application.

### 5.3. Glucose-Responsive Hydrogels

Diabetes mellitus represents a serious global health problem that affects a very large number of people, which highlights the need for effective therapeutic strategies for glycemic control. In type 1 diabetes, insulin administration is mandatory, while in type 2 diabetes, insulin is frequently used in combination with other oral antidiabetic agents, especially in the advanced stages of the disease. Conventional administration via subcutaneous injections does not reproduce the physiological response of the pancreas and is often associated with glycemic fluctuations and discomfort [176].

Glucose-responsive hydrogels are stimuli-responsive polymer networks designed for the encapsulation and release of insulin according to blood glucose levels. Depending on the glucose recognition mechanism, these polymer networks can be developed based on protein systems, such as glucose oxidase (Gox) and concanavalin A (ConA), but they present limitations related to stability and immunogenicity, as well as through non-protein systems, such as phenylboronic acid (PBA), which provides stability and long-term storage, but with a less physiological response compared to enzymatic systems. The most important purpose of glucose-sensitive hydrogels lies in the development of self-regulating systems for insulin administration [177].

Glucose-responsive hydrogels translate variations in glucose concentration into structural changes in the polymer network through a controlled enzymatic and chemical mechanism. Glucose oxidase (Gox) catalyzes the oxidation of glucose to D-gluconolactone in the presence of oxygen, generating gluconic acid and lowering the local pH. Basic groups in the hydrogel become protonated, inducing electrostatic repulsions and promoting swelling of the network and release of the encapsulated insulin. However, accumulation of hydrogen peroxide can inactivate Gox and generate undesirable effects on biocompatibility. To limit this effect, many formulations include catalase, an enzyme that converts hydrogen peroxide into water, thereby maintaining enzymatic activity [176]. Oxygen consumption generates a local hypoxic environment, exploited to trigger insulin release from the hydrogel [178]. In a hypoxic environment, the hydrophobic 2-nitroimidazole group contained in the hydrogel platform is reduced to the hydrophilic 2-aminoimidazole group, leading to changes in the hydrosolubility of the materials and consequently to insulin release [179]. A representative example of a glucose-responsive hydrogel uses a peptide network functionalized with glucose oxidase and catalase for the self-regulated release of insulin [180]. In vitro and in vivo studies have demonstrated that the hydrogel maintains near-physiological glucose levels in diabetic mouse models, highlighting its potential as an effective platform for regulating blood glucose levels.

Glucose-sensitive hydrogels can also be designed using the lectin concanavalin A (ConA), which exhibits selective affinity for monosaccharides, particularly free glucose. At physiological pH, ConA acts as a crosslinking agent for polymers with glycosidic groups, both natural and synthetic, stabilizing the hydrogel network structure through reversible interactions. In the presence of free glucose, the lectin preferentially binds to it, causing relaxation of the network and a gel–sol transition. This structural change promotes increased swelling and controlled release of bioactive molecules, including insulin, proportional to the local glucose concentration [181,182]. Based on these properties, self-regulating devices for insulin delivery exploit glucose-sensitive hydrogels, in which ConA acts as a reversible crosslinking agent: in the presence of glucose, the polymer chains detach from the lectin, reducing the viscosity of the network and activating drug diffusion, and the process reverses as blood glucose decreases. Free glucose competitively occupies the ConA binding sites and induces destabilization and dissociation of the ConA–polymer complexes. Thus, these systems allow rapid and physiological regulation of insulin release [177]. These concepts were confirmed by in vivo tests realized by Taylor et al., which demonstrated the efficacy of an implantable artificial pancreas, made from a biodegradable and biocompatible dextran–concanavalin (Dex/ConA) complex, in diabetic pigs, highlighting a rapid response, precise regulation of release, and the possibility of intramuscular or subcutaneous administration [183].

The glucose-binding properties of concanavalin A were exploited by Lin et al. [184] through the development of a glucose-sensitive hydrogel based on carboxylated pullulan functionalized with concanavalin A, designed for the controlled release of insulin. In this system, ConA is covalently immobilized within the polymer network, maintaining the structural integrity of the hydrogel and preventing the undesired release of the lectin. The specific binding between ConA and glucose induces a reversible modification of the hydrogel network, in which ConA–polysaccharide interactions are competitively disrupted by free glucose, leading to network relaxation and proportional insulin release.

Glucose-sensitive hydrogels based on phenylboronic acid (PBA) exploit the ability of boronated groups to reversibly interact with the cis-diols of carbohydrates. At physiological pH, PBA exists in equilibrium between the neutral trigonal form and the anionic tetrahedral form, the latter binding glucose and generating network swelling, increased porosity, and release of loaded molecules, including insulin. This molecular mechanism confers PBA-functionalized hydrogels the ability to ensure the insulin release proportionally to the local glucose concentration, enabling rapid and reversible self-regulating behavior [185]. The efficiency of the PBA–glucose interaction depends on the pKa of the boronate groups, which controls the proportion between the trigonal form and the anionic tetrahedral species, the only one capable of binding diols. Therefore, optimizing the pKa so that the anionic species is predominant at physiological pH represents an essential criterion in the design of hydrogels with high glucose sensitivity [186]. Phenylboronic acid, classified as a Lewis acid, forms reversible bonds with vicinal diol groups in polysaccharides, conferring hydrogels the ability to respond intelligently to glucose through dynamic adjustment of the polymer network. However, specificity is not exclusive to glucose; other sugars such as fructose and galactose can bind with higher affinities, influencing the selectivity of PBA hydrogels in complex biological environments [187]. In addition to sugars, lactic acid, which is physiologically present in the body, can interact with phenylboronic acids in hydrogels. Under conditions of intense physical exertion, with the generation of elevated lactic acid levels, this interaction may trigger undesirable disturbances in insulin release, highlighting the vulnerability of PBA systems and underscoring the need for the development of optimized platforms [188].

For the application of PBA hydrogels in self-regulated insulin delivery, Lu et al. developed a dissolvable microneedle patch fabricated from PVP, functionalized with 3-fluorophenylboronic acid and amino groups, capable of selectively responding to variations in glucose concentration. The reversible interaction between boronic groups and glucose induces electrostatic changes in the polymer network, enabling proportional insulin release without compromising the structural stability of the hydrogel [189].

Despite stimuli-responsive insulin release, their clinical applicability is limited by constraints specific to glucose recognition mechanisms. Systems based on glucose oxidase are influenced by variations in pH, temperature, and oxygen concentration, and the products of the enzymatic reaction, such as gluconic acid and hydrogen peroxide, can induce toxicity and structural instability, leading to unpredictable or delayed responses [190]. Hydrogels that utilize phenylboronic acid exhibit limited selectivity for glucose due to nonspecific interactions with other biological diols, and the relatively high pKa values require additional chemical modifications for efficient operation at physiological pH [191]. In the case of systems based on concanavalin A, although glucose affinity is high, the use of this lectin is restricted by its immunogenic and cytotoxic potential, as well as by reduced stability in biological environments, which limits in vivo applicability [184]. Overall, these limitations underscore the need to optimize recognition mechanisms and to develop safer and more clinically reliable glucose-responsive systems [177]. However, one of the most significant clinical arguments for stimuli-responsive hydrogels is their potential to reduce dosing frequency—a critical parameter for patient adherence and quality of life. Studies in streptozotocin-induced rat models of type 1 diabetes have demonstrated that glucose-responsive hydrogels (based on phenylboronate or concanavalin A) can maintain glycemia within normal limits for 5–7 days after a single subcutaneous injection, compared with 1–2 daily administrations of conventional insulin [192]. This reduction from daily to weekly dosing (a 7× reduction in frequency) has been replicated in multiple independent laboratories, giving it a reasonable degree of preclinical validity.

### 5.4. Enzyme-Sensitive Hydrogels

Enzyme-responsive hydrogels are materials that are designed to selectively respond to enzyme overexpression in the biological environment. The polymer network is functionalized with specific fragments—polysaccharides, peptides, or amino acids—recognized as substrates by the target enzyme, and local enzymatic reactions, such as hydrolysis of peptide or polysaccharide bonds, induce structural changes in the hydrogel, including swelling or complete degradation. These functionalizations enable controlled drug release and adaptation of the material properties to the biological microenvironment, ensuring precise spatial and temporal control of therapy [193,194,195]. Extracellular proteases are essential enzymes for maintaining tissue homeostasis, extracellular matrix remodeling, and cellular signaling. Under physiological conditions, their levels and activity are finely regulated by specific tissue inhibitors, cytokines, and interactions, maintaining an adequate enzymatic balance.

In pathological contexts, such as neoplasms, chronic inflammation, and degenerative tissue lesions, certain proteases are overexpressed or hyperactivated [194,195,196,197]. Among the most extensively studied are matrix metalloproteinases (MMP-2, MMP-9), hyaluronidase, β-galactosidase, and alkaline phosphatase, each having distinct roles in disease progression [198]. Based on the enzymatic mechanism described above, in which overexpression of target enzymes triggers changes in the hydrogel network and controlled drug release, several hydrogels responsive to these biological stimuli have been developed. For example, an MMP-2-responsive, injectable, in situ-formed hydrogel was designed for the controlled release of doxorubicin and sunitinib nanoparticles, targeting tumors through cleavage of peptide segments sensitive to this enzyme. The elevated MMP-2 activity in the tumor microenvironment triggers localized degradation of the hydrogel, allowing drug release directly in the affected area and reducing tumor recurrence without significant systemic effects [199].

Similarly, an MMP-9-responsive hydrogel, designed for the healing of diabetic wounds, enables controlled release of cinnamaldehyde proportional to enzyme activity. Selective degradation of the hydrogel matrix through peptide cleavage promotes protection of endothelial cells against ferroptosis and stimulates re-epithelialization and angiogenesis, significantly accelerating wound closure in murine models [200]. Vildanova et al. [201] developed hydrogels based on chitosan and pectin, physically crosslinked, for the controlled release of cisplatin. Overexpression of hyaluronidase triggers degradation of the hydrogel matrix and prolonged drug release, highlighting the hydrogel’s potential for enzyme-dependent targeted delivery. Kumar et al. [202] developed an injectable, mucoadhesive, esterase-sensitive hydrogel for targeted delivery of budesonide in experimental ulcerative colitis. Ester bonds in the network, cleaved by esterases, allow controlled drug release directly into the inflamed colonic tissue, alleviating disease severity, restoring colon length, and reducing local inflammatory markers.

Enzyme-sensitive hydrogels exploit the overexpression of enzymes specific to pathological environments, such as matrix metalloproteinases (MMPs), esterases, or proteases, to achieve controlled degradation and targeted drug release. However, their clinical applicability is limited by many factors. A major obstacle is the variability of enzyme expression between patients and between different stages of the disease, which can lead to insufficient or uncontrolled release of the therapeutic agent [194]. In addition, the low specificity of enzymatic substrates can lead to premature degradation of the hydrogel by similar enzymes present in healthy tissues, compromising the selectivity of the system. Another limiting aspect is the difficult control of degradation kinetics, since enzymatic activity is influenced by pH, temperature, inflammation, and the presence of endogenous enzyme inhibitors, which can result in unpredictable in vivo responses [194]. In addition, the hydrogel’s degradation process can generate toxic or reactive compounds, such as hydrogen peroxide generated in enzymatic crosslinking reactions, which can induce local inflammation, cytotoxicity, or tissue necrosis in the areas where the hydrogel is applied [203].

### 5.5. ROS-Sensitive Hydrogels

Reactive oxygen species (ROS)—such as superoxide anion radical, hydrogen peroxide, hydroxyl radical, peroxynitrite, and hypochlorite—are byproducts of cellular metabolism and mitochondrial dysfunction [204,205]. These highly reactive molecules can damage proteins, lipids, and nucleic acids, leading to genetic mutations and cellular dysfunction. Under physiological conditions, ROS levels are maintained within non-toxic ranges through the action of antioxidant systems, such as enzymes (superoxide dismutase, catalase, glutathione peroxidases) and non-enzymatic systems, such as reduced glutathione (GSH), which maintain intracellular redox homeostasis. In various pathologies—such as solid tumors, chronic inflammation, tissue injuries, neurodegenerative diseases, and diabetes—these mechanisms are overwhelmed, leading to excessive ROS accumulation and oxidative stress [204,206,207,208]. ROS-responsive hydrogels exploit redox imbalances in the pathological microenvironment, using oxidation- or reduction-sensitive chemical bonds, such as diselenide (Se–Se) and disulfide (S–S) bonds, which are cleaved in the presence of elevated ROS or increased GSH levels, triggering polymer network disintegration and release of the encapsulated drug [209]. These modifications confer high selectivity and controlled release to the hydrogels, minimizing drug degradation in normal tissues. ROS-responsive materials are designed for local administration and primarily include polymers functionalized with sulfur, selenium, tellurium, or groups such as phenylboronic acid or oxalic acid [204,210].

Clinical and preclinical examples confirm the efficacy of these platforms. Thus, De la Torre et al. synthesized an in situ hyaluronic acid-based hydrogel, loaded with mesoporous silica nanoparticles functionalized with polyethylene glycol and disulfide bonds, carrying Doxorubicin or Safranin O. In the presence of intracellular GSH, the disulfide bonds are cleaved, triggering the opening of the nanoparticle pores and controlled release of the cargo, demonstrating the system’s ability to integrate hydrogel matrix stability with a redox-responsive mechanism [211]. Another relevant example is a ROS-responsive hydrogel designed for chemotherapy, which integrates doxorubicin, a Toll-like receptor agonist, and an anti-PD-1 antibody. The system exhibited ROS level-dependent degradation and controlled release of the therapeutic agents, highlighting the feasibility of combining multiple therapeutic strategies within a single oxidative stress-sensitive hydrogel platform [212]. The use of a diselenide-crosslinked, ROS-sensitive hydrogel has also been reported for multimodal tumor chemotherapy. In this system, local generation of hydrogen peroxide and reduction in diselenide bonds by GSH accelerate polymer network degradation and contribute to the amplification of intracellular oxidative stress, thereby supporting the therapeutic efficacy of the platform [213].

However, ROS-sensitive hydrogels present multiple limitations for clinical applications: variability in ROS expression between patients and conditions affects the control of degradation and drug release, and overexpression or overproduction of ROS can cause redox imbalances and tissue toxicity. In addition, ROS-sensitive linkers must be sufficiently stable at low physiological levels but efficiently release the drug at elevated ROS levels in pathological areas. In tumors with low ROS levels, the incorporation of ROS-generating agents is required, which necessitates precise control of both release and behavior related to these markers [214].

### 5.6. Multiresponsive Hydrogels

Most pathological states are characterized by a complex imbalance of the tissue microenvironment, in which biochemical, physical, and metabolic changes occur simultaneously and interact dynamically. Parameters such as local pH, temperature, reactive oxygen species concentration, enzymatic activity, and metabolic levels, including glucose, undergo significant variations in chronic inflammation, tumors, infected wounds, or metabolic disorders, generating a heterogeneous biological environment that is difficult to address with conventional drug delivery systems [215]. In this context, single-stimulus-responsive hydrogels exhibit intrinsic limitations, with a reduced ability to discriminate between transient physiological variations and therapeutically relevant pathological signals. In response to this complexity, multiresponsive hydrogels have emerged as a natural evolution of stimuli-responsive materials, designed to respond simultaneously or sequentially to two or more internal or external stimuli, adapting more faithfully to real biological environments [216]. By integrating multiple signal recognition and transduction mechanisms within a single polymer network, these systems enable fine spatiotemporal control over physical properties such as swelling, contraction, deformation, or network permeability, as well as drug release. Reversible structural changes, including swelling–relaxation transitions or selective degradation of sensitive bonds, can be exploited to precisely regulate therapeutic release, enhancing treatment specificity and efficacy [217]. A defining advantage of multiresponsive hydrogels lies in their ability to generate synergistic responses, where the combined action of stimuli produces functional effects that exceed the sum of individual responses [3]. A relevant example in cancer therapy is a multiresponsive hydrogel designed to respond to the specific pH of the tumor microenvironment, elevated levels of oxidative stress, and near-infrared (NIR) irradiation. In this system, local acidity and high ROS concentrations induce selective degradation of the sensitive bonds within the polymer network, while NIR irradiation generates local hyperthermia, accelerating drug diffusion. The coordinated action of these stimuli enables controlled and localized release of Doxorubicin, optimizing the therapeutic concentration at the tumor site and reducing systemic side effects [218].

On the other hand, the microenvironment of diabetic wounds, characterized by persistent hyperglycemia, chronic inflammation, infection, and hypoxia, poses a major challenge for the healing process. In this context, multiresponsive hydrogels offer the advantage of simultaneously adapting to multiple pathological local stimuli, enabling the coordinated activation of therapeutic mechanisms according to microenvironmental conditions. Zhou et al. developed a dual-responsive hydrogel that can be activated by both pH and glucose, functionalized with glucose oxidase, catalase, and deferoxamine mesylate, which responds simultaneously to acidity changes and glucose concentrations. This system locally regulates pH and glucose, reduces oxidative stress and hypoxia, modulates inflammation, inhibits bacterial infection, and stimulates angiogenesis, accelerating wound healing in diabetic murine models [219]. Expanding on the concept of multiresponsive hydrogels, another dual-responsive system to pH and ROS was developed by Wu et al. [220] for chronic diabetic wounds. The glycopeptide hydrogel is synthesized from oxidized dextran grafted with phenylboronic acid and ε-polylysine grafted with caffeic acid, featuring self-healing properties and adaptability to the irregular contours of the wound. Sensitivity to acidity and ROS accumulation enables the coordinated release of diclofenac and mangiferin, triggering synergistic therapeutic mechanisms: reduction in inflammation, neutralization of reactive oxygen species, and stimulation of angiogenesis. In vivo models demonstrated accelerated epidermal regeneration, collagen deposition, neovascularization, and reduced local inflammation, highlighting the potential of multiresponsive hydrogels in the complex remodeling of the diabetic wound microenvironment.

In addition to dual-responsive pH/glucose or pH/ROS systems used for applications such as cancer therapy and diabetic wound healing, the literature also reports other multi-stimuli-responsive hydrogels that respond simultaneously to local acidity and elevated temperature, changes characteristic of pathological microenvironments such as inflammation and infected wounds. For example, a system synthesized from N-isopropylacrylamide and acrylic acid, loaded with silver nanoparticles, demonstrated Ag^+^ ion release strictly dependent on pH and local temperature, showing enhanced potency in bacterial elimination and infected wound healing [221]. Other dual-responsive pH/temperature hydrogels were designed for continuous drug delivery, highlighting how variations in pH and temperature within the pathological microenvironment can be exploited for precise control of drug release [222]. Despite their clear advantages, multi-stimuli-responsive hydrogels exhibit significant limitations. The integration of multiple response mechanisms increases synthetic complexity and reduces reproducibility [8]. Furthermore, the precise control of drug release can be compromised by interactions between divergent stimuli, and repeated swelling–deswelling cycles can negatively affect the mechanical stability and durability of the material [3]. These limitations emphasize the need for the development of advanced hydrogel platforms capable of adaptive responses and spatiotemporal control of therapy.

## 6. Advanced Hydrogel Platforms

**Figure 12:**
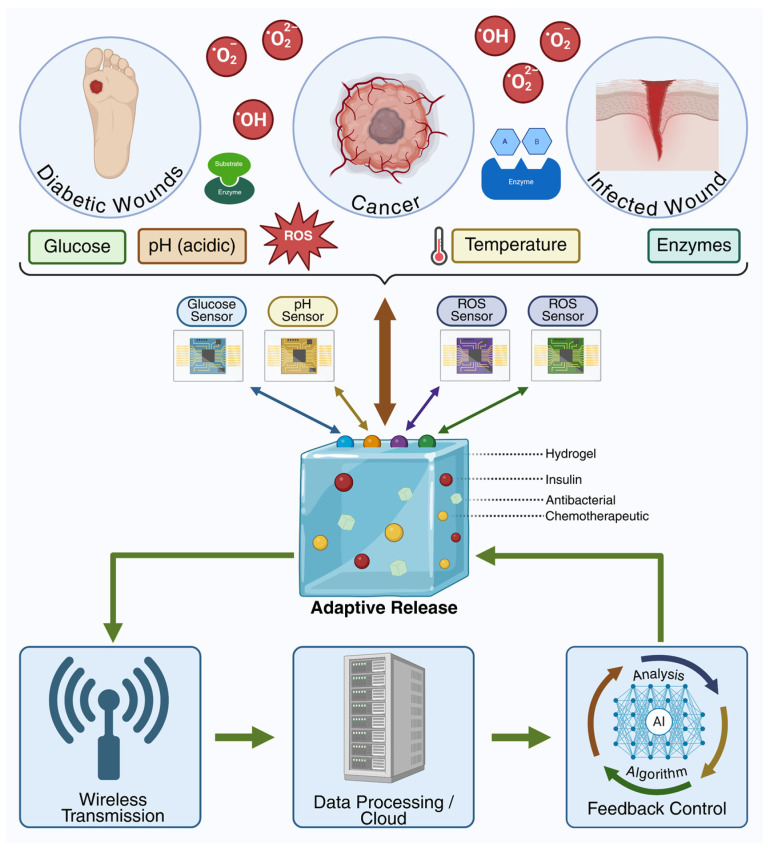
Schematic representation of drug release from advanced hydrogel platforms integrated with biosensors, micro technologies, and AI created in BioRender. (https://BioRender.com/s4hdc0i, accessed on 25 January 2026) is licensed under CC BY 4.0.

Recent advances in hydrogels for drug delivery have surpassed the limits of classical stimuli-responsive systems, leading to the development of sophisticated materials capable of detecting and adaptively responding to local biomarkers. Stimuli-responsive or smart hydrogels, designed to modify their physicochemical properties in response to signals such as pH, temperature, redox, or specific biomolecules, have represented a turning point in targeted therapy and controlled drug release, improving therapeutic efficacy and reducing systemic side effects due to their sensitivity to the specific pathological environment [1]. Smart hydrogels actively respond to local changes through swelling, contraction, or degradation of the polymer network, leading to sustained and targeted release of the therapeutic agent, correlated with the nature and intensity of the external or internal stimulus [156]. In contrast, advanced hydrogel platforms integrate elements detection, such as optical or electrochemical biosensors or other functional structures, which allow real-time identification of local concentrations of pathological biomarkers and dynamic regulation of therapeutic release, proportional to the stimulus level. This direct interaction between the hydrogel matrix and biological signals extends the functionality of the materials from simple stimulus sensitivity to continuous adaptive response [223]. The expansion of the functionality of these platforms includes integration with electronic systems, such as monitoring chips or wireless modules, which allow data transmission to external devices and remote control of drug release, thereby supporting the development of closed-loop feedback systems for personalized administration and continuous patient monitoring (Figure 12) [224].

### 6.1. Hydrogels with Integrated Biosensors for Early Diagnosis of the Pathological Microenvironment

Early detection of biochemical changes in the pathological microenvironment is essential in personalized medicine, as local variations in biomarkers often precede overt clinical manifestations of disease. Integration of biosensors into the hydrogel matrix combines the biocompatibility, selective permeability, and mechanical adaptability of hydrogels with the ability of biosensors to convert biological signals into quantifiable responses, enabling real-time monitoring of pathological parameters and informing therapeutic strategies [225].

Glucose is one of the most studied metabolic biomarkers. A paradigmatic example of a hydrogel-based glucose biosensor is the fluorescent hydrogel optical fiber sensor developed by Li et al., which continuously monitors glucose concentration and pH [226]. The hydrogel fiber is functionalized with phenylboronic acid (3-APBA) and CdTe quantum dots (QDs), converting volumetric and refractive index changes into a fluorescent signal. Simultaneous detection of pH complements its effects on the PBA-glucose reaction, enhancing the accuracy of glucose monitoring.

A flexible electrochemical hydrogel biosensor was developed based on a gel composed of ferrocene-grafted polyethylenimine (PEI Fc), sodium alginate (SA), and PVA, enabling real-time detection of reactive oxygen species (ROS), particularly hydrogen peroxide (H_2_O_2_). The hydrogel structure facilitates differential voltammetry, with a wide detection range (0–120 µM), while its antioxidant properties and biocompatibility make it suitable for monitoring oxidative stress at the tissue level [227]. Additionally, an electrochemical hydrogel system integrated with hydrogelated peptides enabled simultaneous detection of H_2_O_2_ and other reactive species produced by tumor cells in 3D cultures, demonstrating the potential of hydrogels with integrated biosensors for dynamic assessment of oxidative stress and microenvironmental changes associated with tumor progression [25].

Matrix metalloproteinases (MMPs) are valuable biomarkers for the early diagnosis of tumor progression and chronic inflammation. An example of a hydrogel platform for detecting MMP-9 activity is a carboxymethyl chitosan hydrogel functionalized with MMP-9-sensitive peptides, which releases a measurable electrochemical signal proportional to the enzyme concentration, comparable to standard laboratory methods [26]. These examples of hydrogels integrated with biosensors pave the way for advanced platforms in which local biomarker detection is coupled with adaptive drug release, thus transitioning from the realm of detection to that of drug delivery.

### 6.2. Advanced Hydrogel Platforms for Adaptive Drug Release

Effective treatment of rapidly progressing diseases, such as cancer, as well as chronic conditions like diabetes, requires the timely administration of drugs or bioactive biomolecules at optimal doses. Stimuli-responsive hydrogels exhibit rapid changes in response to local stimuli, exhibiting nonlinear feedback behavior and generally returning to their initial form after stimulus removal. However, their ability to continuously adapt to dynamic variations in local biomarkers is limited, especially in complex and fluctuating pathological microenvironments [156]. In this context, advanced hydrogel platforms represent a significant conceptual evolution, as they integrate biosensor detection elements capable of detecting and monitoring real-time changes in biomarker concentrations and regulating drug release in a proportional and adaptive manner. These dynamic systems enable personalized therapeutic interventions based on closed-loop feedback mechanisms, bringing hydrogels closer to the functionality of autonomous treatment systems.

A relevant example in this regard is the glucose-responsive system synthesized from phenylboronic acid-modified gelatin and sodium alginate (AP/SA), designed for local insulin administration in diabetic wounds. The hydrogel operates through a dual detection and release mechanism, in which the phenylboronic groups detect local glucose levels in real time, and changes in concentration trigger proportional insulin release directly at the wound site. Preclinical studies have demonstrated not only efficient glycemic regulation but also stimulation of angiogenesis and local cell proliferation, highlighting the potential of autonomous and adaptive therapy in the management of diabetic wounds [228].

Extending this approach to more complex pathological microenvironments, biosensor hydrogel platforms capable of simultaneously responding to multiple relevant biomarkers have been developed. A recent example is a multi-biomarker hydrogel designed for the treatment of diabetic foot ulcers (DFUs), engineered to detect and adaptively respond to glucose, reactive oxygen species (ROS), and matrix metalloproteinase 9 (MMP-9). The hydrogel was obtained by grafting chitosan with 3-carboxyphenylboronic acid (CS-BA) and integrating epigallocatechin gallate (EGCG) together with oxidized hyaluronic acid, while gelatin microspheres loaded with deferoxamine were used as therapeutic reservoirs. The multilayer architecture of the system allows sequential and adaptive release of therapeutic agents: EGCG is released early to scavenge ROS and reduce inflammation, followed by deferoxamine release in response to elevated MMP-9 levels, stimulating angiogenesis. This closed-loop feedback strategy demonstrates the capability of advanced hydrogels to correlate local biomarker detection with adaptive therapeutic release, optimizing wound healing through modulation of the pathological microenvironment [229]. A complementary approach for adaptive insulin release is represented by glucose self-activated nanozyme-hydrogel platforms, also developed for the treatment of diabetic wounds. In these systems, glucose oxidase (Gox) is used as the detection element, and insulin as the therapeutic agent, both incorporated into bimetallic Zn-Fe nanoparticles with a metal–organic framework structure (MOF(Zn-Fe)/Gox/INS), dispersed in a ROS-sensitive hydrogel. In hyperglycemic conditions, Gox catalyzes the oxidation of glucose, generating gluconic acid and hydrogen peroxide, which induces local acidification and degradation of the MOF structures, leading to controlled insulin release. Upon restoration of normoglycemia, the catalytic activity automatically stops, preventing the risk of hypoglycemia.

This feedback-based closed-loop detection and adaptive release mechanism is associated with increased therapeutic efficacy, antibacterial effects, and stimulation of tissue regeneration, and has also been reported in silk fibroin-based hydrogels functionalized with phenylboronic acid [230]. The principles of adaptive release are also applied in the oncological context, where the tumor microenvironment is characterized by increased acidity and overexpression of matrix metalloproteinases. In this regard, MMP-2-sensitive hydrogels were designed for targeted delivery of chemotherapeutic agents. A relevant example is an injectable hyaluronic acid-based hydrogel, crosslinked with MMP-2-sensitive peptides and loaded with biodegradable micelles containing doxorubicin. In the presence of MMP-2 activity in the tumor tissue, the hydrogel selectively degrades, resulting in local and sustained release of doxorubicin and inhibition of tumor growth in preclinical squamous cell carcinoma models [231]. This functional transition toward hydrogels with adaptive release governed by biomolecular feedback lays the groundwork for their inclusion in wearable devices and smart microtechnologies, ensuring continuous monitoring and real-time therapeutic control.

### 6.3. Hydrogels Integrated with Wearable Devices and Smart Microtechnologies

In recent years, research in the field of hydrogels integrated with microtechnologies has advanced beyond simple monitoring and stimuli-responsive release toward wearable bioelectronic platforms capable of wireless control and adaptive decision-making. In this context, these advanced hydrogel platforms, often referred to in the literature as functional hydrogels, integrate hydrophilic networks loaded with therapeutic agents with biosensors, flexible microelectronics, and wireless communication systems, enabling continuous monitoring of the biological microenvironment and adaptive regulation of drug release [232].

A representative example of integrating hydrogels with smart technology was reported by Du et al. The authors developed a wireless, programmable, and refillable hydrogel bioelectronic system to accelerate the healing of diabetic wounds. The device combines a conductive hydrogel matrix with a flexible circuit equipped with a microcontroller and Bluetooth modules for wireless control of drug release and local electrostimulation. The hydrogel, composed of chitosan and PVA with graphene oxide as a conductive filler, allows diffusion of the loaded drug and responds to preset electrical signals, so that release profiles can be adjusted via wireless signals according to therapeutic needs [233]. In vitro tests proved that the drug release rate can be controlled by varying the applied voltage, and in vivo experiments in diabetic mice confirmed the system’s efficacy in accelerating wound healing, highlighting the potential of wearable bioelectronic hydrogels to combine storage and therapeutic delivery functions within a single treatment platform.

Expanding the concept of wearable hydrogels with precise control, a recent example utilizes a drug reservoir made of sulfonated poly(3,4-ethylenedioxythiophene) (PEDOT) polyelectrolyte hydrogel, capable of responding simultaneously to endogenous stimuli (body temperature) and exogenous stimuli (electrical stimulation) for on-demand transdermal delivery. Functioning both as a drug reservoir and as the cathode in a Zn battery-powered iontophoresis patch, this hydrogel enables controlled release of dexamethasone (≈12.2 μg cm^−2^ over 3 h at 37 °C), facilitating the treatment of atopic dermatitis. This wearable platform highlights the efficiency of dual-responsive hydrogels and their relevance for precise, non-invasive drug administration [234]. Demonstrating a new level of technological integration, Deng et al. [235] developed a flexible, wireless hydrogel dressing that combines biomarker monitoring with adaptive therapeutic release. The hydrogel, functionalized with matrix metalloproteinase-9 (MMP-9)-sensitive peptides, is connected to an LC radiofrequency sensor, enabling real-time assessment of enzyme levels in chronic wounds. In response to the detected signal, the hydrogel releases silver nanoparticles (AgNPs) in a controlled manner, providing local antimicrobial effects. This platform illustrates the potential of smart hydrogels integrated with microtechnologies to support personalized treatment strategies and continuous monitoring.

Nanoparticles have revolutionized the field of biosensors through their unique ability to amplify signals and provide increased molecular specificity. Functionalization with targeting ligands allows for precise detection of biomarkers at extremely low concentrations, opening new perspectives in early diagnosis [236].

Chemical and physical compatibility between components is essential for efficient integration but remains underexplored in the current scientific literature. Recent studies demonstrate that optimizing interfaces through covalent immobilization methods significantly reduces nanoparticle losses and increases sensor durability. Hybrid macromolecule–nanoparticle systems represent a promising frontier, combining the mechanical properties of polymers with the optical and electronic characteristics of nanoparticles [237]. Densities of 10–20 ligands per polymer chain represent the optimal threshold for maximizing multivalency, according to recent research in nanomedicine. This configuration allows simultaneous binding to multiple target sites, dramatically increasing affinity and specificity for biological structures. The multivalency effect reduces nonspecific interferences and increases the overall sensitivity of the system.

FDA-approved nanoparticle–polymer hybrid wound dressing systems demonstrate the translational success of these technologies. These medical devices combine functionalized silver or gold nanoparticles with biocompatible polymers, providing controlled release of antimicrobial agents and promoting tissue healing [238].

**Figure 13:**
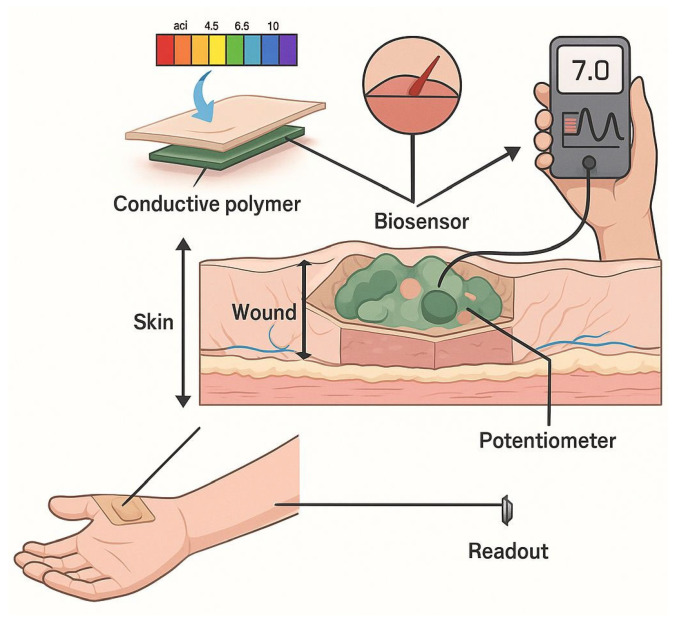
Schematic representation of the electrochemical biosensor system used for real-time monitoring of wound pH. Reprinted from Nicolae A.M. et al., 2025 [223], *Biosensors* [CC BY 4.0, https://doi.org/10.3390/bios15120773].

Recent studies describe smart wearable systems that use hydrogels integrated into wearable electronic devices, capable of monitoring physiological signals and delivering therapy adaptively, tailoring treatment according to the patient’s condition and the dynamic requirements of the microenvironment [239]. The integration of AI becomes essential for analyzing large volumes of complex data and recognizing subtle patterns associated with disease progression (Figure 13), transforming hydrogels from stimulus-responsive systems into predictive and decision-making platforms [223,240].

In this context, a recent clinical study reported the development of a wearable hydrogel patch integrating multiple SERS biosensors and AI algorithms for monitoring the progression of lung cancer [27]. The hydrogel functions as an active matrix for sweat absorption, while the biosensors enable simultaneous detection of multiple biomarkers. AI-driven data analysis facilitated real-time assessment of therapeutic response and the identification of disease stages.

These advances outline a new framework in which hydrogels evolve from simple stimulus-responsive delivery systems into interactive, predictive, and integrative platforms, supporting real-time clinical decision-making, monitoring disease dynamics, and evaluating therapeutic responses, thereby paving the way for high-precision therapies [241].

Despite significant progress, advanced hydrogels integrated with biosensors, microtechnologies, and AI face numerous challenges. Biosensor reliability can be limited by signal instability and material degradation in dynamic biological environments, restricting the sensitivity and specificity of real-time detection. Integrating microtechnologies with the hydrogel matrix requires optimization of the material–electronic interface and the maintenance of stable connections between flexible electronic components and the soft material, posing critical challenges for the performance of wearable systems [242,243,244]. Wireless data transmission is affected by factors such as signal attenuation in biological fluids, the energy consumption of communication modules, and the need for compact and efficient power sources, limiting continuous communication without compromising biocompatibility [245,246]. Moreover, integrating AI algorithms for interpreting complex data raises issues related to the need for large volumes of labeled data and the robustness of models against biological variability, posing a challenge for the validation and generalization of smart systems in diverse clinical contexts [247].

These challenges reflect the current limitations of advanced hydrogel platforms in the context of smart therapies. Nevertheless, ongoing optimization of functional materials, integrated microtechnologies, and AI-based analytical strategies could, in the future, facilitate the clinical translation and scalability of these complex hydrogel platforms.

## 7. The Main Administration Routes of Hydrogels

**Figure 14:**
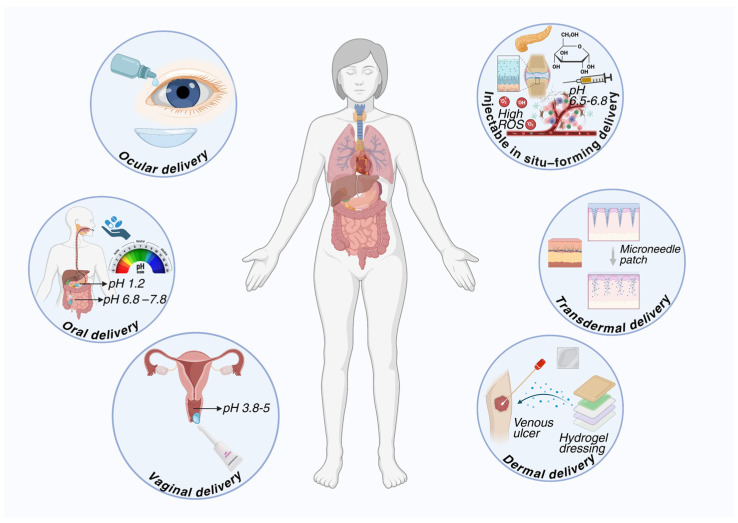
Schematic overview of the main administration routes of hydrogels created in BioRender. (https://BioRender.com/ptgxw15, accessed on 13 March 2026) is licensed under CC BY 4.0.

Hydrogels, due to their versatile physicochemical properties and tunable structures, can be administered via multiple routes to achieve both local and systemic therapeutic effects. Depending on the target tissue, desired release profile, and nature of the encapsulated bioactive agents, hydrogels can be delivered orally, through injection, via mucosal surfaces, or across the skin (Figure 14). Each administration route offers distinct advantages and challenges, influencing drug stability, bioavailability, and patient compliance, while their careful selection is crucial for optimizing therapeutic outcomes.

### 7.1. Oral Administration

Gastrointestinal drug administration remains one of the most widely used non-invasive treatment methods due to ease of application. The extensive surface area and rich vascularization of the gastrointestinal mucosa allow for efficient drug absorption, which guarantees a representative potential for both systemic and local oral therapies. However, the effectiveness of administration can be influenced by physiological factors such as the acidic pH of gastric juice, digestive enzymes, rapid transit time, and the mucus layer lining the epithelium, all of which affect drug stability, retention, and absorption.

In this context, polymeric hydrogels have sparked interest as advanced platforms for oral drug delivery due to their mucoadhesive properties and ability to provide controlled release, maintaining therapeutic molecules in close proximity to the mucosa and protecting them from enzymatic and chemical degradation [248]. For gastric applications, hydrogels are often designed to gel in situ, forming stable three-dimensional networks that promote local retention and targeted release [249]. For example, Rehman et al. developed hybrid chitosan–alginate hydrogel spheres loaded with graphene oxide conjugated to folic acid and doxorubicin, designed for controlled release at slightly acidic pH (~5.5), characteristic of tumor microenvironments. Ex vivo tests demonstrated high mucoadhesivity resulting from electrostatic interactions between chitosan and gastric mucus, while in vitro studies showed gradual drug release over 24 h. In vivo evaluations in healthy rabbits confirmed the retention and functionality of the hybrid polymeric matrix, highlighting the potential of gastroretentive hydrogels to improve the efficacy of local therapy in gastric cancer [250]. The same principles are applicable to the treatment of gastric infections. In *Helicobacter pylori* infections, conventional antibiotic therapies are limited by the need for high doses, degradation in the acidic gastric environment, and the emergence of bacterial resistance. Controlled local delivery of antibiotics allows dose reduction and enhanced therapeutic efficacy [251]. A recent example of a gastro-retentive system for *H. pylori* infections is hybrid alginate/PEGDA hydrogel films, designed to combine prolonged gastric retention with local antibiotic release. These films exhibit shape-memory properties, allowing compression into a capsule for oral administration and rapid re-expansion in gastric juice (≥70% recovery in ~4 min), ensuring prolonged retention in the stomach throughout therapy. Mechanical properties (20–80 kPa) provide resistance to peristaltic forces, maintaining structural integrity during gastric residence. Antibacterial activity against *E. coli*, *S. aureus*, and *H. pylori* was maintained for over 24 h [252].

As drug delivery systems transit into the small intestine, physiological conditions change significantly. The higher pH, particularly in distal segments, the thin and discontinuous mucus layer, the extensive surface area provided by villi, and relatively constant motility, create a favorable environment for controlled drug absorption. These features support the use of pH-sensitive hydrogels for the release of active substances at the intestinal level. However, intense enzymatic degradation, especially in the duodenum, and epithelial barriers can limit bioavailability, highlighting the need for advanced platforms capable of protecting sensitive molecules [249]. This challenge is particularly relevant for protein and peptide molecules, such as insulin, which are highly susceptible to acidic and enzymatic degradation. Oral insulin delivery is extensively investigated due to its potential to improve patient compliance and better mimic physiological pancreatic secretion [253]. Wu et al. proposed a system based on alginate hydrogel incorporating liposomes loaded with an arginine–insulin complex, capable of reducing premature release and enhancing intestinal absorption. The system demonstrated effective protection in the gastric environment, increased intestinal permeability ex vivo, and a significant in vivo hypoglycemic effect, confirming the role of hydrogels as versatile matrices for oral delivery of sensitive biomolecules [254].

Targeted colonic delivery represents a critical approach for the treatment of a wide range of local disorders, such as colorectal cancer, inflammatory bowel diseases, Crohn’s disease, and ulcerative colitis. Due to its distal location, colon-targeted systems must prevent premature release in proximal segments and ensure controlled activation at the colonic site. The neutral to slightly alkaline pH, reduced motility, and prolonged retention time make the colon a favorable environment for hydrogels sensitive to local conditions [255]. Illustrating this approach, Raben et al. developed a hydrogel film with dual responsiveness to pH and colonic enzymes, enabling selective release of metronidazole and mesalamine in the colonic environment and demonstrating spatiotemporal control of drug delivery [256].

In colorectal cancer, the characteristics of the tumor microenvironment, including slightly acidic pH, accumulation of reactive oxygen species (ROS), overexpression of matrix metalloproteinases, and specific enzymatic activity of the colonic microbiota, have been exploited in the design of smart hydrogels capable of controlled disintegration and enhanced local release of chemotherapeutic agents. Responsiveness to these intrinsic stimuli facilitates controlled degradation of the system and regulated release of chemotherapeutics [257,258,259,260]. In a recent study, Li et al. developed an oral inulin-based hydrogel incorporating hollow MnO_2_ nanocarriers loaded with oxaliplatin, designed for targeted colonic delivery of this chemotherapeutic. The system protects the drug during gastrointestinal transit and triggers selective release in the colon via microbiota-mediated degradation, demonstrating significant in vivo antitumor effects. The incorporation of MnO_2_ further amplifies tumor oxidative stress, enhancing the cytotoxic effect of oxaliplatin and highlighting the critical role of hydrogel design in the efficacy of localized oral therapy for colorectal cancer [261].

Overall, these studies demonstrate that the rational adaptation of hydrogel structure and composition to the physiological characteristics of each segment of the gastrointestinal tract enables spatiotemporally controlled drug release, increased local retention, and protection of sensitive molecules. This underscores the role of hydrogels as polymeric platforms for targeted oral delivery in both acute and chronic gastrointestinal mucosal disorders. However, the clinical translation of oral hydrogels remains limited by inter- and intra-individual physiological variability of the gastrointestinal tract, differences in motility, mucus composition, and microbiota, as well as challenges related to scaling, reproducibility, and precise control of the released dose under real administration conditions.

### 7.2. Injectable Route

Injectable hydrogels have evolved a lot in recent years in the field of drug delivery, offering benefits such as controlled and targeted release, formation of local depots, and mechanical adaptability to host tissue [262]. Unlike preformed hydrogels, which require surgical implantation and may deform under mechanical stress, injectable hydrogels can be administered directly at the target site. These include stimulus-sensitive pre-gels that undergo in situ sol–gel transition and shear-thinning hydrogels capable of passing through a syringe needle and rapidly reforming their polymer network. The integration of reversible physical bonds (hydrogen bonds, hydrophobic interactions) and dynamic covalent bonds (Schiff base interactions and disulfide bonds) provides structural stability and self-healing functionality, making hydrogels versatile platforms for controlled drug delivery [263].

Formulation strategies play a crucial role in controlling gelation and the release of therapeutic agents at the target site. Thermoresponsive polymers, such as PNIPAM, undergo a sol-to-gel transition at body temperature, ensuring injectability and rapid formation of a stable local depot [264,265]. Ionotropic polysaccharides, like alginate, form stable networks through crosslinking with divalent cations, maintaining gel integrity under physiological conditions [96,266,267]. Additionally, chemically or enzymatically crosslinked hydrogels, including those made from gelatin or fibrin, solidify in situ through local reactions, allowing precise tuning of mechanical properties and release kinetics [203,268,269]. By combining the intrinsic properties of polymers with these formulation strategies, injectable hydrogels can adapt to irregular anatomical spaces, form local depots, and provide sustained drug release, reinforcing their potential as versatile therapeutic platforms.

Injectable hydrogels have proven effective in the treatment of numerous pathological conditions, including oncological disorders [16], chronic inflammatory diseases such as rheumatoid arthritis [270], diabetes [271], as well as in the management of pain associated with various pathological states [272].

In oncology, the tumor microenvironment—characterized by acidic pH, hypoxia, oxidative stress, and elevated enzymatic activity—can be leveraged through stimuli-responsive hydrogels, enabling local and controlled drug delivery with rapid formation of an intratumoral depot and reduced systemic exposure [273,274,275]. In vitro and in vivo studies have demonstrated the efficacy of injectable hydrogels in delivering chemotherapeutics [276], immunotherapeutic agents [277], and gene therapy vectors [278], supporting their utility in managing various cancer types. A representative example of an intelligent injectable hydrogel for localized chemotherapy delivery is a chitosan-based system combined with the PNIPAAm-co-itaconic acid (IA) copolymer, crosslinked with glycerophosphate [279]. This hydrogel responds simultaneously to multiple stimuli, including low pH (~5.5) and slightly elevated temperature (>37 °C), typical of the tumor microenvironment. In vitro studies showed sustained doxorubicin release over 8 days without burst release, maintaining high therapeutic concentrations. The incorporation of itaconic acid was critical for controlling swelling and release, highlighting the importance of smart crosslinking in designing injectable anticancer hydrogels. Injectable hydrogels can also preserve the bioactivity of monoclonal antibodies, facilitating controlled release and minimizing adverse effects associated with conventional administration [280]. In persistent pain management, injectable hydrogels—including peptide-based, thermosensitive, and nanocomposite systems—have proven effective in delivering opioids and local anesthetics, extending analgesic effects from several days up to a week in preclinical models [281,282,283].

An efficient strategy involves the use of injectable hydrogels in inflammatory joint diseases, such as rheumatoid arthritis, where synovial inflammation and chronic pain are difficult to control with systemic therapies. By maintaining therapeutic concentrations directly at the joint, these systems reduce the systemic toxicity associated with conventionally administered anti-inflammatory drugs and DMARDs [284,285,286]. These platforms target specific pathogenic mechanisms, including oxidative stress, secretion of pro-inflammatory cytokines such as TNF-α and IL-1β, activity of matrix metalloproteinases, and HIF-1α–mediated imbalances in the synovial microenvironment [287]. Recent studies highlight the potential of thermosensitive and stimuli-responsive hydrogels for controlled intra-articular delivery of anti-inflammatory and immunomodulatory agents, demonstrating reduced inflammation, prolonged therapeutic effects, and decreased injection frequency [270,288,289]. Beyond primarily local applications, injectable hydrogels are expanding toward systemic therapies. In particular, diabetes mellitus—a metabolic disorder—requires glycemic control predominantly mediated by peptide-based biologics, such as GLP-1 receptor agonists, which play a key role in regulating blood glucose levels and promoting weight reduction [290].

Injectable hydrogels represent a promising strategy for the delivery of antidiabetic biologics, providing protection against enzymatic degradation and enabling controlled, prolonged release. This approach can reduce injection frequency and improve patient adherence [271]. In this context, supramolecular PNP hydrogels and thermoresponsive PLGA–PEG–PLGA hydrogels have been developed, demonstrating sustained in vivo release of encapsulated GLP-1 agonists, maintaining therapeutic plasma concentrations for several weeks to over a month [271,291,292]. Similarly, a chemically crosslinked silica-based injectable hydrogel enabled extended release of pramlintide, maintaining therapeutic plasma levels for up to 60 days in diabetic mouse models [293]. However, their clinical translation is limited by tissue microenvironment variability, restricted injectable volumes, risk of local reactions, and challenges in large-scale reproducibility. Moreover, the lack of clinical data on immunological safety and long-term sustained release highlights the need for integrated, rigorous studies to validate their predictable benefits in medical practice [12,271,291,292].

### 7.3. Transmucosal Administration

**Figure 15:**
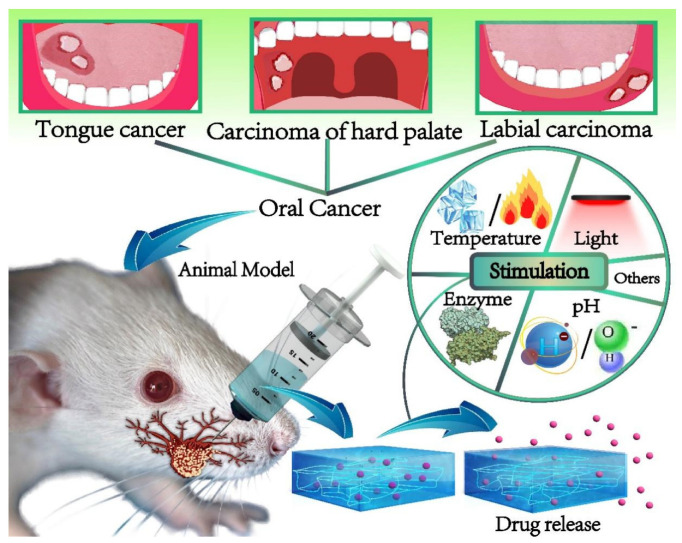
Smart hydrogel-based drug delivery system for oral cancer therapy. Reprinted from Zhao Y. et al., 2022 [297], *Gels* [CC BY 4.0, https://doi.org/10.3390/gels8110741].

Transmucosal administration offers a valuable alternative to conventional drug delivery routes by bypassing first-pass metabolism and avoiding gastrointestinal degradation, while also reducing the discomfort associated with injections. Mucosal surfaces—oral, nasal, ocular, and vaginal—provide accessible entry points that can enable both local and systemic drug absorption. However, delivering drugs via the mucosa presents several challenges. These are largely due to the unique characteristics of mucosal tissues, which can limit drug absorption and promote rapid clearance, as well as factors related to patient adherence, such as the difficulty of maintaining the formulation in place for sufficient durations [19,294,295]. In this context, transmucosal hydrogels have emerged as versatile drug delivery platforms due to their mucoadhesive properties and controlled-release capabilities. They prolong contact with the mucosal surface, enhancing local retention, and protect therapeutic agents from premature degradation, improving efficacy and stability [296]. At the oral level, these hydrogels enable local retention of the formulation and prolonged, controlled release of the active agent, making them suitable for localized treatment of oral cancer (Figure 15) [297].

A recent example is the hydrogel developed by Zaheer et al., based on cyclodextrin and thiolated hydroxyethyl cellulose, designed for local delivery of cisplatin [298]. This formulation allowed efficient drug loading, maintained contact with the oral mucosa for extended periods, and provided controlled release, highlighting the potential of this platform for targeted oral cancer therapy.

The nasal route provides rapid access to the systemic circulation and enables direct delivery to the central nervous system (CNS), without crossing the blood–brain barrier—a major limitation of conventional administration [299]. However, physiological factors of the nasal mucosa, such as mucociliary clearance, nasal secretions, and the epithelial barrier, can limit mucosal residence time and drug bioavailability. In this context, nasal hydrogels, due to their mucoadhesive properties and polymeric architecture, enhance local retention, protect drugs from enzymatic degradation, and allow controlled release of active compounds, increasing residence time and therapeutic efficacy [300]. For instance, Su et al. [301] developed a thermosensitive chitosan-based nasal hydrogel combined with PEG–PLA nanoparticles, for local delivery of miRNA-146a, in the treatment of allergic rhinitis. The formulation rapidly forms a mucoadhesive gel and provides sustained release of the therapeutic load. Preclinical studies showed a significant reduction in nasal inflammation and superior efficacy compared to conventional formulations, confirming the potential of nasal hydrogels for local delivery of biologics. While most nasal hydrogel formulations are developed for local effects, recent studies demonstrate their potential for systemic administration and CNS delivery. For example, Pina Costa et al. [302] developed an in situ hydrogel incorporating nanostructured lipid carriers (NLCs) loaded with diazepam, designed for nasal administration to facilitate brain delivery in epilepsy treatment. The hydrogel was administered as an aerosol via a multidose pump optimized for nasal delivery. Deposition was evaluated using a 3D-printed human nasal cavity model, demonstrating proper gelation, high mucoadhesion, and prolonged diazepam release.

In ophthalmic applications, hydrogels have been studied for their local action, where conventional methods face significant limitations due to the physiological barriers of the eye, such as rapid tear drainage, the corneal barrier, and natural self-cleaning mechanisms, which reduce retention time and drug bioavailability [300,303]. The clinical relevance of these systems is demonstrated both by patents and clinical studies, particularly in the treatment of dry eye syndrome [304]. In addition, hydrogel applications extend to other ocular conditions, such as inflammation and post-surgical pain, exemplified by the intracanalicular insert based on polyethylene glycol (PEG) hydrogel, DEXTENZA^®^, which enables sustained release of dexamethasone for a period of 30 days [305]. Such polymeric platforms are also being investigated in preclinical studies for a wide range of ocular pathologies, both infectious and non-infectious, highlighting their potential as versatile local drug delivery systems [306]. It is well known that the use of hydrogels in ophthalmology originated in the early 1960s, with the synthesis by Wichterle and Lim of poly(2-hydroxyethyl methacrylate) (pHEMA), a material that formed the basis for the development of the first soft contact lens [307]. This discovery established hydrogels as reference biomaterials in ocular applications due to their favorable combination of optical transparency, oxygen permeability, and biocompatibility. Beyond their role in correcting refractive errors, polymeric hydrogel contact lenses are extensively investigated as ophthalmic drug delivery systems, benefiting from the polymer network architecture, which ensures the sustained release of therapeutic compounds in various ocular pathologies [308].

For example, hydrogel lenses containing graphene oxide as a functional material, loaded with cyclosporine, have demonstrated a significant increase in ocular residence time, allowing prolonged drug release and, consequently, enhanced local bioavailability, while maintaining ocular safety and comfort [309].

Recent developments have extended the functionality of hydrogel contact lenses beyond their role as passive drug-release matrices, through the integration of biosensors and wireless communication modules capable of real-time monitoring of tear biomarkers. The information obtained can be leveraged to dynamically adjust the drug release profile, shaping adaptive and personalized therapeutic strategies. In this context, smart contact lenses are emerging as multifunctional polymeric platforms, reinforcing the role of hydrogels as key materials for the next-generation ocular drug delivery systems [310,311,312,313].

Hydrogels have also been explored for administration via the vaginal mucosa, an attractive alternative route due to the extensive mucosal surface, increased permeability, and rich vascularization, which allow local therapeutic effects and selective systemic absorption through the uterine first-pass effect. This route facilitates targeted delivery of drugs to the uterus, avoiding systemic circulation and degradation associated with oral administration, while its noninvasive nature supports clinical applicability [314]. However, the vaginal environment represents a dynamic and complex biological system that influences formulation performance: the stratified epithelium and mucus layer control permeability and interaction with the hydrogel, and the acidic pH, enzymatic activity, and local microbiota can affect drug stability and bioavailability [315]. In addition, continuous secretions, self-cleaning mechanisms, and mucosal changes associated with hormonal variations throughout the menstrual cycle limit the retention time and effectiveness of conventional formulations [316]. Due to their intrinsic properties, hydrogels offer a promising strategy to overcome these challenges.

Mucoadhesiveness and the ability to respond to local stimuli, such as pH and temperature, allow prolonged retention of hydrogels and controlled drug release, optimizing therapeutic efficacy and reducing the need for frequent administrations [317,318]. Mucoadhesive and thermoresponsive hydrogels have demonstrated effectiveness in the local delivery of antibacterial and antifungal agents, as well as compounds with systemic effects [319,320,321]. For example, a mucoadhesive hydrogel composed of methacrylated chitosan, hyaluronic acid, and PNIPAAm enabled prolonged release of progesterone and retention of the formulation on the vaginal mucosa, highlighting the potential of hydrogels as efficient platforms for local administration to achieve systemic effects [322].

Although transmucosal hydrogels have shown potential in prolonging retention and demonstrated efficacy due to mucoadhesiveness and responsiveness to local stimuli, the physiological variability of the vaginal environment and the limited number of in vivo studies still constrain their clinical applicability. Optimizing formulations through careful selection of polymers and design strategies remains essential to achieve prolonged retention on the vaginal mucosa and efficient delivery of therapeutic molecules [315,317,318,323].

Although transmucosal hydrogels have demonstrated remarkable potential in prolonging retention and controlling drug release, their clinical applicability remains limited. Physiological factors specific to each mucosa—such as secretions, epithelial barriers, mucus dynamics, and pH variations—affect bioavailability and therapeutic efficacy. Moreover, most studies are limited to in vitro models or a small number of in vivo studies, reducing the ability to extrapolate results to real clinical conditions. To fully harness the potential of mucoadhesive hydrogels, intelligent design strategies, careful selection of mucoadhesive and stimuli-responsive polymers, and formulation optimization are necessary, particularly to improve local retention, which remains constrained.

### 7.4. Cutaneous and Transdermal Route

Cutaneous drug administration has become an important therapeutic strategy with the development of modern delivery systems, due to its non-invasive nature, increased patient compliance, and avoidance of limitations associated with oral or injectable administration [324]. However, the efficiency of conventional formulations, such as creams, ointments, or traditional gels, is severely limited by the skin’s barrier function, particularly the stratum corneum, which restricts the permeation of most therapeutic molecules [325]. In this context, the use of smart polymeric materials, such as hydrogels, has attracted considerable interest, providing a versatile platform for dermal and transdermal drug delivery [326]. Hydrogels have demonstrated effectiveness in local dermatological therapies, such as inflammatory dermatitis, by retaining active substances at the site of application and optimizing the release profile [327,328]. For example, a hydrogel loaded with tretinoin and clindamycin led to a significant reduction in acne lesions and exhibited good cutaneous tolerability, validating this platform as an effective therapeutic option [329]. In addition, the efficacy of dermal hydrogels has been evaluated in pain management. A comprehensive review of randomized clinical studies reported the use of hydrogels for the topical administration of analgesic drugs, demonstrating significant improvement in pain control and good local tolerability [272].

**Figure 16:**
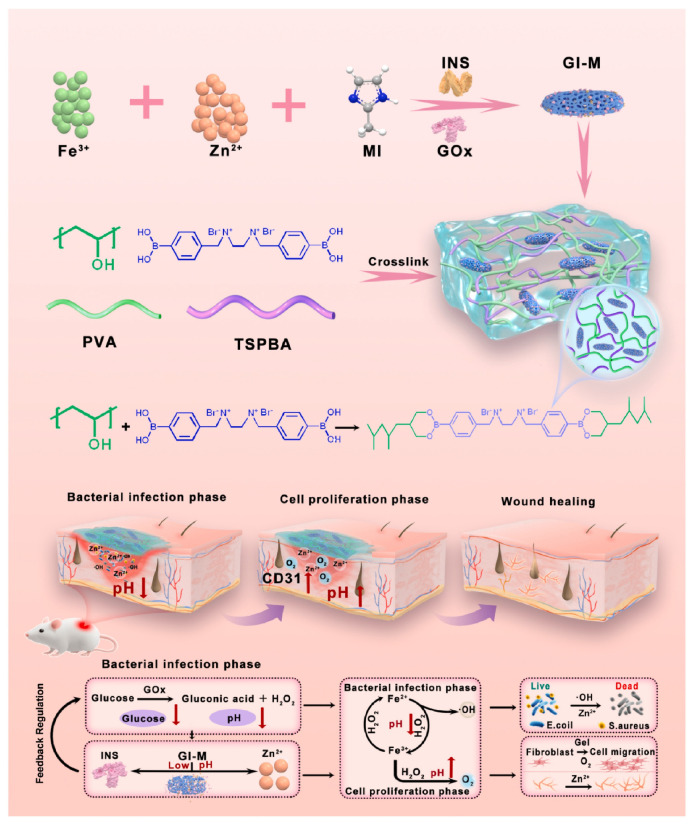
Schematic representation of the multifunctional hydrogel with cascade enzyme-mimetic activity for the treatment of infected wounds in diabetes. Reprinted from Tai Q.D et al., 2025 [230], *Materials Today Bio* [CC BY-NC-ND 4.0, https://doi.org/10.1016/j.mtbio.2025.102405].

Beyond conventional dermatological applications, hydrogels have an important potential in wound treatment, where the integrity of the skin barrier is compromised and the risk of persistent infection and inflammation is elevated [330]. These lesions, which include venous ulcers, diabetic ulcers, pressure sores, and burns, are characterized by delayed or disrupted physiological healing processes and are often refractory to conventional therapies [331]. One of the main factors complicating wound healing is infection, as the presence of pathogens induces significant changes in the wound microenvironment, including local pH variations, increased temperature, accumulation of reactive oxygen species, and overexpression of infection-specific enzymes, all associated with persistent local inflammation [332]. In this context, hydrogels sensitive to pH, ROS, or bacterial enzymes (Figure 16) enable controlled release of antimicrobial or anti-inflammatory agents, exclusively under active infection conditions, thereby reducing unnecessary antibiotic exposure and the risk of antimicrobial resistance [230,333].

A relevant example of an intelligent hydrogel used for the treatment of severely infected burns is the self-healing hydrogel composed of quaternized chitosan and oxidized dextran, loaded with tobramycin and polypyrrole nanofibers coated with polydopamine (QCS/OD/TOB/PPY@PDA), developed by Huang and colleagues [334]. The system utilizes dynamic Schiff base bonds, enabling pH-sensitive antibiotic release that is accelerated in the acidic microenvironment induced by infection. The hydrogel demonstrated sustained antibacterial activity against resistant pathogens, including *Pseudomonas aeruginosa*, *Staphylococcus aureus*, and *Escherichia coli*, for up to 11 days, while simultaneously reducing inflammation and promoting in vivo tissue regeneration, leading to improved healing of infected burns.

Diabetic wounds represent major complications of diabetes mellitus and are characterized by a complex microenvironment that is highly unfavorable for healing, defined by local hyperglycemia, persistent inflammation associated with the overexpression of proinflammatory cytokines (particularly TNF-α and IL-6), elevated oxidative stress, dysregulated enzymatic activity, and variations in local pH—all factors that promote chronicity of the wound [335,336]. In this context, hydrogels have been proposed as innovative therapeutic platforms capable of supporting tissue regeneration and providing controlled delivery of therapeutic agents [337]. For example, Chen and colleagues [338] synthesized a multifunctional, injectable, self-healing, and adhesive hydrogel based on polyvinyl alcohol (PVA) and a boronic linker (TSPBA), integrating gelatin microgels loaded with sodium fusidate and nanoliposomes containing metformin. The system demonstrated remarkable antibacterial activity (>98% eradication of MSSA and MRSA within 24 h), local regulation of glucose levels for over 15 days, and efficient neutralization of reactive oxygen species, leading to complete healing of infected diabetic wounds in approximately 14 days in murine models.

Transdermal drug delivery via hydrogels is of particular importance due to its non-invasive nature, ability to maintain steady plasma concentrations, and potential for systemic delivery [339]. These properties have been harnessed through the development of hydrogel-based transdermal patches. Bashyal and colleagues reported a PVA–PVP hydrogel patch for controlled systemic delivery of donepezil, which demonstrated sustained in vitro drug flux, enhanced by the inclusion of propylene glycol as a permeability enhancer. In vivo studies confirmed systemic absorption and prolonged release, achieving plasma concentrations comparable to those obtained after oral administration [340]. Despite these advances, hydrogel patches still face limitations related to the skin barrier, drug stability, and incomplete control over release, which can negatively impact systemic absorption and, consequently, drug bioavailability [341].

To overcome the limitations imposed by the skin barrier, hydrogel-forming microneedles represent an advanced extension of transdermal patches, enabling penetration of the stratum corneum without leaving residues in the skin. These microneedles, made from biocompatible crosslinked polymers, swell rapidly after application, forming stable channels for sustained drug release. Their composition and crosslinking density allow fine-tuning of the release kinetics [342]. In this context, hydrogel microneedles have attracted particular interest for systemic insulin delivery. Thus, Chen and colleagues developed glucose-responsive microneedles based on silk fibroin and a semi-interpenetrating network of boronate-acrylamide copolymer, capable of autonomously releasing insulin according to local glucose levels, demonstrating efficient adaptive glycemic control [343]. Complementarily, Zhu et al. proposed a hybrid microneedle patch combining a rapidly dissolving tip with a swellable hydrogel backing, allowing initial fast release followed by sustained delivery. In vivo studies in diabetic animal models showed a significant reduction in blood glucose within approximately 3 h, maintained within a safe range for up to 6 h [344].

Despite these promising results, hydrogel microneedles still face limitations related to drug loading capacity, dose-release variability, stability of sensitive molecules, and challenges associated with manufacturing and regulatory requirements, which may hinder clinical translation [345]. To overcome these obstacles, intelligent transdermal closed-loop systems have been developed, integrating hydrogel microneedles with electrochemical glucose biosensors and adaptive release mechanisms, enabling automatic and personalized insulin administration according to blood glucose levels [346].

## 8. Conclusions and Future Perspectives

As research on smart hydrogels advances, their future platforms are increasingly shaping up as an interdisciplinary convergence between materials chemistry, bioelectronic engineering, computer science, and personalized medicine. Clearly, the transition from proof-of-concept demonstrations to scalable and robust clinical applications represents the key challenge in the coming years. In this context, theranostic hydrogels emerge as a promising multifunctional platform that integrates diagnostic and therapeutic capabilities within a single material system [347]. These advanced hydrogels enable real-time monitoring of tissue status while delivering controlled therapeutic agents, combining antimicrobial protection, tissue regeneration support, and responsive drug release. Integration of biosensors, nanomaterials, and stimuli-responsive polymers enhances their adaptive performance and multifunctionality. Despite their advantages—biocompatibility, mechanical robustness, and customizable design—challenges remain regarding clinical efficacy, device compatibility, and long-term performance. Future developments are expected to focus on nanoscale material optimization, integration with real-time detection technologies, and application of artificial intelligence to further improve precision, personalization, and therapeutic outcomes. Theranostic hydrogels therefore represent a key frontier in next-generation biomedical technologies, bridging the gap between diagnosis and treatment in personalized healthcare. The integration of hydrogels in wearable devices and flexible bioelectronics offers a pathway for continuous monitoring and personalized therapeutic intervention. Functionalized hydrogels can act as soft, biocompatible, and adaptable elements on detachable or wearable platforms, providing real-time detection of physiological signals and the ability to communicate with external systems for therapy control and adjustment. Such strategies aim to overcome the limitations of conventional monitoring systems through hydrogel-type materials that combine biocompatibility with elasticity and signal conduction, required for advanced wearable functionality.

An exciting prospect for the future is CRISPR gel technology, which represents a convergence between materials engineering and molecular biology, opening new horizons for personalized medicine and precision gene therapies. The ability of these systems to maintain the integrity of CRISPR components in complex biological environments and to release their content at the optimal time and place fundamentally transforms the approach to treating genetic diseases.

In addition, the transition to sustainable polymers in biomedical applications is not only an ecological necessity but also an opportunity for technological innovation. Traditional polymers such as PEG are gradually being replaced by bio-based alternatives such as polylactide (PLA), cellulose and chitosan, which come from renewable sources and are biodegradable, thus contributing to the circular economy.

Also, in the future, preclinical validation using microfluidic platforms such as organ-on-chip, which reproduce the physiological functions of human organs, will provide more relevant models than animal tests. These biomimetic devices integrate cell cultures, micrometric fluidics and mechanical stimuli to recreate the natural tissue microenvironment, allowing the observation of cellular responses in real time. In the era of advanced biotechnology, preclinical validation stands at the intersection of genetic innovation, ecological sustainability, and precise physiological modeling. Together, these technologies promise to reduce development time and costs, improve the safety and efficacy of therapies, and minimize environmental impact.

On the other hand, the need is emerging to integrate hydrogels with artificial intelligence (AI) and the Internet of Things (IoT) to move from reactive systems to predictive platforms. AI can play a crucial role in interpreting the large volumes of data generated by networks of hydrogel biosensors, facilitating the recognition of subtle patterns associated with disease progression, anticipating therapeutic responses, and automatically optimizing the drug release profile according to the patient’s clinical evolution. This approach —where the system detects, analyzes, and acts autonomously—is identified as a key direction for the development of personalized therapies that dynamically respond to pathological signals. In addition, microfluidics and advanced printing (3D/4D) will have a major impact on future generations of smart hydrogels. The use of microchannels to control biofluid flows and to mimic complex physiological environments allows for efficient testing and optimization of hydrogels in near-clinical scenarios prior to deployment. Hydrogels used as soft interfaces between tissue and electronic hardware can significantly enhance patient comfort, while simultaneously raising challenges related to the material–electronic interface, signal durability, and long-term safety in use. However, aspects related to sustainability, biocompatibility, and regulation must not be neglected, as they will play an increasingly important role as these technologies expand their applicability. Standardization of testing, large-scale reproducibility, long-term evaluation, and integration of safety and data privacy requirements are parameters that will shape the clinical adoption of smart hydrogels. Thus, the future of smart hydrogels is oriented toward enhanced connectivity, AI-based analysis, and advanced microtechnologies, supporting treatments that are more precise, more efficient, and more closely aligned with the individual needs of patients.

## Figures and Tables

**Table 1:** Materials Used for Hydrogel Synthesis as Smart Polymeric Platforms for Drug Delivery.

Material	Class	Relevant Properties for Drug Delivery Systems (DDSs)	Type of Response/Functionality	Typical Applications in Drug Delivery	Main Limitations	Ref.
**Chitosan**	Natural Polysaccharide	Cationic, Mucoadhesive Biodegradable Antibacterial activity, Biocompatible	pH-responsive, Thermo-responsive, Paracellular permeability	Controlled oral administration (proteins, peptides, insulin, anticancer drugs), Injectable, Wound healing	Low solubility at neutral pH, limited mechanical strength, Low thermal stability, high batch-to-batch variability	[101]
**Alginate**	Natural Polysaccharide	Anionic, ionic gelation (“egg-box”), Biocompatible	pH-dependent swelling, Sustained release, Ionic strength	Oral administration, Colon-targeted DDS, In situ injectable	Non-enzymatically biodegradable, low mechanical stability, low mechanical strength	[102,103,104]
**Pectin**	Natural Polysaccharide	Anionic, degree-of-methoxylation-dependent gelation, Biocompatible, Biodegradable Stable in acidic environment	pH- and enzyme-responsive (colonic microbiota), Mucoadhesive, High water absorption	Oral administration, Colon-targeted DDS, Platform for antibacterial and anticancer formulations	Modest mechanical strength, structural variability, Limited control over release kinetics	[105]
**Hyaluronic acid**	Natural Polysaccharide	Anionic, bioactive, high water retention, CD44 affinity, Biocompatible, Biodegradable hydrophilic, and viscoelastic	Enzyme (hyaluronidase), ROS- and pH-responsive	Targeted delivery (anticancer), Oral, in situ injectable, transdermal	Accelerated enzymatic degradation in vivo, low mechanical stability	[106,107]
**Collagen/gelatin**	Natural Structural Proteins	Biocompatibility, biodegradability, high porosity, thermo-/chemically gelable, RGD-mediated cell adhesion	Enzyme- and ROS-responsive, in situ gelation	Oral, ocular, in situ injectable administration, DDS for oncological therapy	Structural variability, rapid enzymatic degradation, modest mechanical strength	[108,109]
**DNA**	Nucleic acid (natural biopolymer)	Biocompatibility, biodegradability, complementary sequence programmability, porosity, tunable multifunctionality	pH-responsive, thermo-responsive, sol–gel transitions, programmed volume changes	DDS, biosensing, protein- or nucleic-acid-based therapeutic systems	Uncertain biological stability, uncontrolled drug release, undesired immune response, insufficient in vivo monitoring, reduced predictability, poor reproducibility	[110,111]
**RNA**	Nucleic acid (natural biopolymer)	Biocompatible, biodegradable	pH-responsive, enzyme-responsive, thermo-responsive	Drug release (DDS), gene therapy, vaccine delivery, antitumor therapy	Enzymatic instability, rapid degradation, limited release control, need for vectors, complex formulation, low stability	[112]
**Lignin**	Natural	Biocompatibility, bioregenerability, amorphous, anionic, porous structure	Hydrophilicity, antioxidant, antimicrobial	DDS, tissue scaffold, wound dressings	Requires chemical modification for functionalization, uneven release, structural variability, difficult standardization	[113,114]
**Poly(ethyleneglycol) (PEG)**	Synthetic Polymer	Biocompatible, water-soluble, non-toxic, adjustable molecular weights, easily derivatizable, biologically inert, precise crosslinking control	Controlled diffusion; stimulus-responsive platform	Injectable, Nanocarriers, Protein delivery	Lack of intrinsic bioactivity, potential systemic effects	[115,116]
**Poly(vinylalcohol)(PVA)**	Synthetic Polymer	Hydrophilic, structural stability, hydrogen bonding	Swelling-controlled release; glucose-responsive platform	Oral administration, Smart insulin delivery systems	Low biodegradability	[117,118]
**Polyurethane**	Synthetic Polymer	Biocompatible tunable mechanical properties, block-segmented polymer structure	Thermosensitivity; pH-responsive in specific derivatives	Controlled transdermal release, Active dressings	Potential cytotoxicity of diisocyanates Top of Form Bottom of Form	[119,120,121]
**Poly(N-isopropylacrylamide) (PNIPAm)**	Synthetic Polymer	Critical temperature (LCST) approx. 32 °C	Thermo-responsive, dual response (pH and temperature)	Injectable systems, On-demand DDS	Limited biodegradability, modest loading capacity	[122,123]

Note: Green column denotes natural polymers; blue column denotes synthetic polymers; and yellow highlights the column presenting typical hydrogel applications in drug delivery.

**Table 2:** Comparative Table: Key Properties of Individual Polymers vs. Hybrid Systems.

Material/Form	Swelling Ratio (%)	Degradation Rate	Mechanical Properties (kPa)	Biocompatibility	Release Efficiency (%)	Ref.
**Chitosan**	200–400	Slow–Moderate (enzymatic)	1–10	++++	60–80	[101,134,135]
**Chitosan/Alginate Hybrid**	300–600	Moderate (tunable)	5–30	++++	70–90	
**Alginate**	300–800	Slow (non-enzymatic)	0.5–5	++++	55–75	[102,103,104,136,137]
**Alginate/Pectin** **Hybrid**	400–900	Moderate (enzyme + pH)	2–15	++++	65–85	
**Pectin**	400–1000	Moderate (colonic enzymes)	0.5–8	++++	60–80	[105,138,139]
**Pectin/Chitosan Hybrid**	350–800	Moderate–Slow (tunable)	5–20	++++	70–88	
**Hyaluronic acid (HA)**	500–2000	Fast (hyaluronidase)	0.1–2	++++	50–70	[106,107,140,141]
**HA/Collagen** **Hybrid**	600–1800	Moderate (tunable crosslinking)	2–20	++++	65–85	
**Collagen/** **gelatin**	300–700	Fast (collagenase/MMP)	0.5–5	++++	55–75	[108,109,142,143]
**Collagen/PEG Hybrid**	400–900	Moderate (crosslink- dependent)	5–50	++++	70–90	
**Poly** **(ethylene** **glycol) (PEG)**	100–500	Very slow (hydrolytic)	1–100 (tunable)	++++	60–85	[115,116,144,145]
**PEG/PNIPAm Hybrid**	200–600	Slow–Moderate	5–80	+++	75–95	
**Poly(vinylalcohol)(PVA)**	100–300	Very slow (non-biodegradable)	10–200	+++	50–70	[117,118,146,147]
**PVA/** **Chitosan** **Hybrid**	200–500	Slow–Moderate (partial)	15–150	++++	65–85	
**Polyurethane**	50–200	Slow (hydrolytic/oxidative)	100–10,000	++	40–65	[119,120,121,148]
**Polyurethane/PEG** **Hybrid**	100–350	Moderate (tunable)	50–5000	+++	55–80	
**PNIPAm**	100–400	Slow (non-biodegradable)	1–20	++	50–70	[122,123,149,150]
**PNIPAm/Alginate Hybrid**	200–600	Moderate (ionic + thermal)	5–40	+++	70–90	
**DNA**	500–1000	Tunable via cross-linking	800–1200	++++	50–90	[110,111,112,151,152]
**Chitosan/Alginate mRNA Hybrid**	300–650	Slow	50–200	++++	42	
**Lignin/PVA/Chitosan**	540–900	Slow	900	+++	40–95	[153,154]

Note: ++++ = excellent, +++ = good, ++ = moderate, + = poor.

## Data Availability

No new data were created or analyzed in this study.

## Abbreviations

The following abbreviations are used in this manuscript:

PLGA	Poly(lactic-co-glycolic acid)
PEGDA	Poly(ethylene glycol) diacrylate
PEG	Poly(ethylene glycol)
CS	Chitosan
DN	Double Network
UDP	Uridine Diphosphate
HA	Hyaluronic Acid
PEGDMA	Poly(ethylene glycol) dimethacrylate
PCL	Polycaprolactone
PLA	Polylactic Acid
PVA	Poly(vinyl alcohol)
PNIPAm	Poly(N-isopropylacrylamide)
PDEAm	Poly(N,N-diethylacrylamide)
PU	Polyurethane
CMCS	Carboxymethyl Chitosan
MAA	Methacrylic Acid
PVP	Polyvinylpyrrolidone
GO	Graphene Oxide
TNT	Titanium Nanotubes
AgNPs	Silver Nanoparticles
IPN	Interpenetrating Polymer Network
MMP_2_	Matrix Metalloproteinase-2
MMP_9_	Matrix Metalloproteinase-9
siRNA	Small Interfering RNA
mRNA	Messenger RNA
PGE_2_	Prostaglandin E2
TNFα	Tumor Necrosis Factor-alpha
GoX	Glucose Oxidase
PBA	Phenylboronic Acid
ConA	Concanavalin A
GC	Glycated Chitosan
LCST	Lower Critical Solution Temperature
UCST	Upper Critical Solution Temperature
VEGF	Vascular Endothelial Growth Factor
shRNA	Short Hairpin RNA
GMS	Glyceryl Monostearate
MPO	Myeloperoxidase
SOD	Superoxide Dismutase
GSH	Glutathione
ADA	Dialdehyde Alginate
OCMC	O-Carboxymethyl Chitosan
IFNγ	Interferon-gamma
PLM	Persistent Luminescent Material
AR	Rheumatoid Arthritis
DMARD	Disease-Modifying Antirheumatic Drug
NSAIDs	Nonsteroidal Anti-Inflammatory Drugs
COX-2	Cyclooxygenase-2
RA GLP-1	Glucagon-Like Peptide-1 Receptor Agonist
RHAMM	Receptor for Hyaluronic Acid-Mediated Motility
LYVE-1	Lymphatic Vessel Endothelial Hyaluronic Acid Receptor 1
TLR	Toll-Like Receptor
ROS	Reactive Oxygen Species
